# The Thyroid Under Pressure: Heavy Metals, Endocrine Disruptors and Translational Insights into Carcinogenesis and Thyroid Dysfunctions

**DOI:** 10.3390/ijms27125583

**Published:** 2026-06-20

**Authors:** Marco Capezzone, Gabriella Pellegriti, Anna Ronchi, Fiorenza Gianì, Andrea Corsello, Rosa Maria Paragliola

**Affiliations:** 1 UOSD of Endocrinology, Misericordia Hospital, I-58100 Grosseto, Italy; 2 Medical Oncology, Department of Clinical and Experimental Medicine, University of Catania, I-95123 Catania, Italy; 3 Pavia Poison Control Centre—National Toxicology Information Centre—Clinical and Experimental Lab, Toxicology Unit, Istituti Clinici Scientifici Maugeri IRCCS, I-2700 Pavia, Italy; 4 Department of Medicine and Surgery, “Kore” University, I-94100 Enna, Italy; 5 Unit of Endocrine Surgery, Ospedale Isola Tiberina-Gemelli Isola, I-00186 Rome, Italy; 6 Departmental Faculty of Medicine, UniCamillus-Saint Camillus International University of Health Sciences, I-00131 Rome, Italy

**Keywords:** thyroid, endocrine-disrupting chemicals, heavy metals, thyroid cancer, thyroid dysfunction, environmental pollutants

## Abstract

The thyroid gland is particularly vulnerable to the effects of environmental pollutants, due to its high vascularization, dependence on iodine uptake, and intrinsic oxidative environment required for hormone biosynthesis. Therefore, environmental exposure to heavy metals (HMs) and endocrine-disrupting chemicals (EDCs) has emerged as a potential contributor to thyroid dysfunction and carcinogenesis. Despite increasing interest, the clinical relevance of these exposures remains incompletely defined. Available epidemiological data suggest heterogeneous associations across EDCs and HMs classes. While evidence is more consistent for some pollutants, for other compounds it remains limited. Furthermore, while experimental studies provide strong mechanistic support for the key pathways linking environmental exposure to thyroid dysfunction and carcinogenesis, the clinical interpretation of epidemiological data is constrained by important methodological limitations. This narrative review aims to integrate current epidemiological and experimental evidence on the role of HMs and EDCs in thyroid diseases, including both non-neoplastic disorders and thyroid cancer, examining their environmental distribution, exposure pathways, and biological effects.

## 1. Introduction

Thyroid carcinoma (TC) represents the most common endocrine malignancy and currently ranks as the 7th most prevalent cancer worldwide [1]. Over the past five decades, its incidence has risen significantly, increasing from 5.0 cases per 100,000 people in 1975 to 14.6 cases per 100,000 people in 2009 [2,3]. After this marked increase, incidence rates plateaued and, in several high-income countries, have shown a decline in recent years. This is especially true for small TC, and it is probably an effect of the adoption of recent guidelines’ recommendations discouraging fine needle aspiration of nodules <1 cm [4]. Importantly, despite these changes in incidence, TC-specific mortality remained stable, supporting the notion that overdiagnosis, due to the widespread use of diagnostic imaging, has played a significant role [5]. Notably, data from the United States indicate a modest but significant increase in TC mortality, the underlying causes of which are still unclear [6]. Papillary thyroid cancer (PTC) accounts for about 90% of all TC cases [7] and is generally associated with an excellent prognosis. Within PTC, some histological subtypes are associated with more aggressive behavior, including tall cell, diffuse sclerosing, columnar cell, hobnail, and solid subtypes.

Although the incidental detection of small, indolent TC due to overdiagnosis accounts for much of the historical rise in incidence, accumulating evidence suggests that environmental factors may contribute to this trend [8]. Beyond well-established risk factors such as exposure to ionizing radiation, female sex, and inherited genetic mutations [9], recent studies have emphasized the potential role of obesity and exposure to environmental factors, including endocrine-disrupting chemicals (EDCs) and heavy metals (HMs), in the pathogenesis of TC [8] The marked geographical heterogeneity detected in TC incidence suggests a role for environmental factors. In Italy, one of the European countries with the highest TC incidence, pronounced regional differences have been reported, with increased risk observed in areas characterized by specific geological features and documented HM contamination [10], including volcanic regions [11].

Furthermore, environmental factors may not only exert direct carcinogenic effects but also induce thyroid dysfunctions, including hypothyroidism and autoimmune thyroid diseases. Notably, both autoimmune thyroid diseases [12] and hypothyroidism can be considered additional risk factors for TC, particularly considering evidence that higher serum TSH levels are associated with an increased risk of malignancy in thyroid nodules, as well as with more advanced-stage TC [13].

Despite increasing experimental evidence, the effects of HMs and EDCs on thyroid function and thyroid carcinogenesis are still not fully understood, particularly due to the limited availability of robust data in humans. In fact, animal and in vitro models are frequently evaluated separately from clinical and epidemiological studies, limiting translational interpretation.

This narrative review is based on a comprehensive and critical evaluation of the current literature addressing the relationship between environmental pollutants and thyroid diseases (thyroid dysfunction and thyroid carcinogenesis) with particular focus on HMs and EDCs. Epidemiological studies, experimental in vitro and in vivo models, meta-analyses, and relevant reports from scientific and regulatory agencies were integrated to provide a broad overview of this field. Furthermore, particular attention was devoted to the molecular mechanisms of thyroid dysfunctions and carcinogenesis, aiming to integrate mechanistic findings with clinical and epidemiological observations. Considering the marked heterogeneity of the literature, the present work was conceived as a narrative review. In this paper, HMs and classical EDCs are considered as distinct, yet partially overlapping categories. HMs are defined based on their chemical nature as inorganic elements characterized by environmental persistence and bioaccumulation, whereas EDCs represent a functional class of exogenous compounds defined by their ability to interfere with hormone action. Notably, several HMs have been shown to exert endocrine-disrupting effects, particularly at the level of thyroid function. Therefore, the distinction adopted in this review is primarily based on chemical classification. Pharmaceuticals, endogenous hormones, naturally occurring dietary compounds, and inorganic iodide uptake inhibitors are outside the scope of the present review.

For easier consultation, the review has been structured into four major sections, summarized in the roadmap presented below. Section 2 provides an overview of the principal classes of HMs and EDCs, including their environmental distribution, classification, and main routes of human exposure. Section 3 focuses on the association between HMs and EDCs and non-neoplastic thyroid diseases, particularly thyroid dysfunction and autoimmunity. Section 4 examines the available evidence linking environmental exposure to thyroid carcinogenesis. Finally, the Section 5 discusses the principal molecular and cellular mechanisms involved in thyroid disruption and carcinogenesis.

Roadmap of the review

- Section 2. Heavy metals and endocrine disruptors: classification, environmental distribution, and exposure pathways
- Section 3. Environmental exposure and non-neoplastic thyroid diseases
- Section 4. Thyroid carcinogenesis: epidemiological and experimental evidence
- Section 5. Molecular mechanisms, endocrine disruption, and translational insights
- Conclusions and future perspectives

## 2. Heavy Metals and Endocrine Disruptors: Classification and Exposure

### 2.1. Heavy Metals: Overview

The term “heavy metal” (HM) is commonly used to describe metallic chemical elements and metalloids characterized by high environmental persistence and which may exert toxic effects on the environment and humans. From a toxicological point of view, these elements can be broadly classified into essential metals, such as copper (Cu), zinc (Zn), and manganese (Mn), which are required for physiological functions (see section below) but become toxic at elevated concentrations, and non-essential metals, including cadmium (Cd), lead (Pb), and mercury (Hg), which have no known biological role and are toxic even at low levels. It is important to underline that, throughout this review, the term “heavy metals” will be used in a broader toxicological sense, encompassing not only non-essential metals but also essential trace elements when present at concentrations exceeding physiological levels and exerting toxic effects.

HMs are naturally occurring components of the Earth’s crust and are persistent environmental pollutants. They are defined as “heavy metals” either due to their high atomic weight or because of their high density (>4 g/cm^3^). Metalloids tend to form covalent bonds with organic groups. Hence, they form lipophilic ions and compounds, and they can generate toxic effects when they bind to non-metallic elements of cellular macromolecules [14]. Metals cannot be broken down and are nonbiodegradable. Organisms may detoxify metal ions by hiding the active element within a protein or depositing them in intracellular granules in an insoluble form. The toxicity and carcinogenicity of HMs are dose-dependent [15], and simultaneous exposure to more HMs may have cumulative effects [16].

The International Agency for Research on Cancer (IARC) has classified HMs into four groups:

*Group 1*—carcinogenic to humans (Arsenic (As) and inorganic compounds, Cd and Cd compounds, Chromium (Cr) VI compounds, Nickel (Ni) compounds, Beryllium and Beryllium compounds, Aluminum production),

*Group 2*—probably/possibly carcinogenic to humans (Pb compounds inorganic and Pb, Vanadium pentoxide, Methylmercury, Ni metallic and alloys, Cobalt, Molybdenum trioxide),

*Group 3*—not classifiable as to carcinogenicity in humans (Cr III compounds, Cr metallic compounds, Hg and inorganic Hg compounds, Se and Se compounds, Cu),

*Group 4*—probably not carcinogenic to humans (Mn, Silver, Zn).

In addition to their intrinsic physicochemical properties, the health impact of HMs is strongly influenced by their sources and pathways of human exposure. HMs are released into the environment through both natural and anthropogenic processes, leading to their widespread distribution in soil, water, air, and vegetation. Natural sources include rock weathering, volcanic activity, marine aerosols, and wildfires [17,18]. Anthropogenic activities represent the main contributor to current environmental contamination, particularly through agriculture (fertilizers, pesticides), industrial processes such as mining and metal refining, fossil fuel combustion, and urban emissions, including wastewater discharge and waste incineration [17,19,20].

#### 2.1.1. Essential Trace Elements and Thyroid Homeostasis

Normal thyroid function requires adequate nutritional levels of iodine, tyrosine, and certain trace elements, including essential metals such as Cu, Zn, iron (Fe), and Mn, as well as selenium (Se), an essential trace element. These elements play essential roles in hormone synthesis, antioxidant activity, and maintenance of thyroid homeostasis.

Cu is involved in tyrosine metabolism and regulates thyroid activity as an antagonist in cases of hyperthyroidism [21]. Furthermore, it exerts antioxidant function and supports cellular metabolism and the immune system [22].

Zn is a component of the enzyme superoxide dismutase, and it is involved in thyroid hormone synthesis and thyroid function regulation [23]. Interestingly, a nutritional survey conducted in patients with medullary TC (MTC) found higher Zn concentrations in the MTC groups compared to healthy controls [24].

Fe is an essential cofactor for thyroid peroxidase (TPO) activity, and it is involved in thyroid hormone biosynthesis [25]. Fe deficiency has been associated with impaired thyroid hormone production, altered thyroid metabolism, and increased risk of hypothyroidism, especially in pregnant women and children [26]. Furthermore, Fe deficiency may reduce the efficacy of iodine supplementation programs in iodine-deficient areas [27].

Mn is involved in antioxidant activity and immune system regulation, and may contribute to the maintenance of thyroid cellular homeostasis. It has been hypothesized that Mn may convert carcinogens into metabolites that are more easily excreted and may improve detoxification capacity in the liver [28].

Se is a trace element essential for thyroid homeostasis [29], as it is a cofactor of selenoproteins, including glutathione peroxidase, protecting the thyroid from oxidative damage. An inverse association has been reported between Se levels and TC risk, particularly PTC [30]. Se deficiency is associated with impaired thyroid hormone synthesis as well as structural thyroid damage [31].

#### 2.1.2. Heavy Metals: Sources of Exposure for the Human Body

Human exposure to HMs occurs mainly through inhalation, ingestion of contaminated food or water, and, to a lesser extent, dermal contact. Soil represents one of the main HM reservoirs, with contamination occurring from waste disposal, sewage sludge discharge, agricultural practices, and industrial emissions [32]. Due to their resistance to degradation, HMs can persist in soils and be transferred to plants and crops [32]. Accordingly, plants can be used as bioindicators of environmental contamination, and elevated HM concentrations have been reported in vegetation from areas characterized by natural or anthropogenic emissions, including volcanic regions [33]. Dietary exposure represents a major pathway for HM human intake. The HMs released into the environment may accumulate in food crops and aquatic organisms, resulting in biomagnification along the food chain [34,35]. Certain foods, including leafy vegetables, fruits, rice, and seafood, have a particularly high potential for HM accumulation [36].

Water contamination is another important route of exposure. Metals released from industrial, urban, and agricultural sources can persist in aquatic environments and accumulate in sediments and marine organisms [37,38,39,40,41]. Airborne exposure may also occur through particulate matter originating from both natural and anthropogenic sources [42]. In addition, occupational exposure represents a relevant pathway in industrial settings concerning metal production and processing [43]. Overall, the persistence of HMs in environmental matrices and their capacity for bioaccumulation contribute to chronic human exposure [44]. Following inhalation or ingestion, HMs can bioaccumulate and cause systemic complications. HMs disrupt cellular homeostasis, interfering with growth, proliferation, differentiation, damage-repairing processes, and apoptosis, and may induce epigenetic alterations leading to genomic instability. A central mechanism of toxicity is the induction of oxidative stress through increased reactive oxygen species (ROS) production, impairment of antioxidant defenses, and enzyme inactivation. Although the present review focuses on thyroid-specific effects, it is important to contextualize these within the broader spectrum of systemic damage induced by HMs, as summarized in Table 1.

Early-life exposure represents a critical contribution to HM toxicity [67]. Exposure during organogenesis can cause permanent changes in structure and anatomy, while if it occurs in the final stages of pregnancy, it can cause functional alterations [68]. During pregnancy, the fetus is particularly vulnerable to HMs, as the placental barrier, although partially protective, does not completely prevent the transfer of several HMs, including Hg, Pb, and, to a lesser extent, Cd. Indeed, these contaminants have been detected in placental tissue, amniotic fluid, and umbilical cord blood [69]. Prenatal and early postnatal exposure, including through breastfeeding, has been associated with adverse developmental, neurological, and endocrine outcomes [70], supporting the concept that early-life environmental exposures may contribute to long-term disease susceptibility.

#### 2.1.3. Heavy Metals: Environmental Distribution, Exposure Pathways and Toxicokinetics

**Figure 1:**
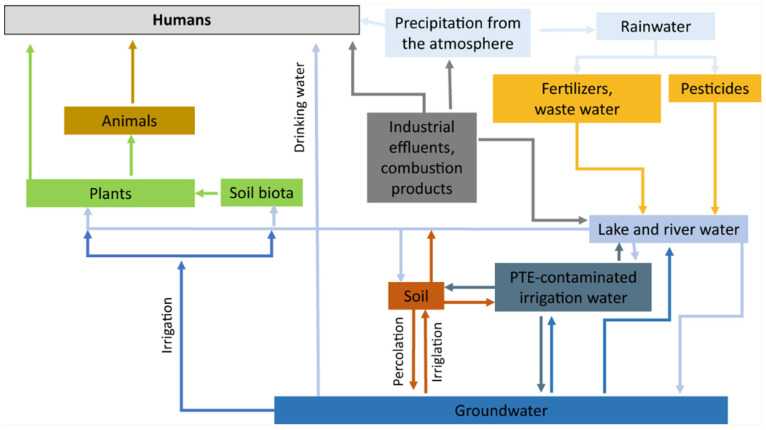
The figure reports a schematic overview of the main environmental sources of heavy metals (HMs) and of principal pathways of human exposure. Natural and anthropogenic sources contribute to contamination of air, soil, and water, with subsequent entry into the food chain. Human exposure occurs mainly through ingestion, inhalation, and, to a lesser extent, dermal contact. The persistence and bioaccumulation of HMs favor chronic low-dose exposure and potential endocrine effects. The arrows indicate the possible directions of movement and transfer of potentially toxic elements among environmental, biological, and human compartments.

In the next sections, several heavy metals that have been most consistently implicated in thyroid dysfunction and related disorders, including Cd, As, Hg, Pb, Cr, and Mn, will be discussed, focusing on their main sources, environmental distribution, and pathways of human exposure. Figure 1 summarizes the major environmental sources of HMs and their main routes of human exposure, highlighting the complexity of real-life exposure scenarios.

*Cadmium (Cd)*

Cd is naturally present in the Earth’s crust, together with Zn, Pb, and Cu ores, and it is present at low concentrations in surface and marine waters, but anthropogenic activities and the use of phosphate fertilizers and sewage sludge contribute to environmental contamination [71]. Cd is widely used in industrial applications, including nickel–cadmium batteries, pigments, coatings, plastic stabilizers, non-ferrous alloys, photovoltaic devices, and specialized manufacturing processes [72,73].

Once released, Cd contaminates multiple environmental matrices, including air, soil, vegetation, and water [71]. In soils, Cd is bioavailable to plants, facilitating its entry into the food chain [74]. In aquatic systems, Cd shows a relatively long persistence, promoting bioaccumulation in biota [75]. For the general population, dietary intake (cereals, leafy vegetables, root crops, and seafood) and tobacco smoke represent primary routes of exposure [71,76]. Occupational exposure is mainly observed in industrial settings [71]. Cd is absorbed through inhalation and gastrointestinal routes and accumulates primarily in the liver and kidneys [77]. Its biological half-life ranges from 5 to 30 years, resulting in progressive accumulation over time [78].

*Arsenic (As)*

It is a chemical element classified as a metalloid, as it exhibits properties intermediate between those typical of metals and non-metals. From a toxicological perspective, it is commonly grouped with HMs due to its high toxicity and ability to bioaccumulate. It can exist in four oxidation states. All forms of As are considered toxic, but the degree of toxicity depends on mobility, chemical speciation, and the ability to interact with endogenous compounds [79].

As originates from both natural (volcanic activity, rock weathering) [18] and anthropogenic sources, including mining, fossil fuel combustion, industrial processes, and pesticide use. Due to its high mobility, As can contaminate groundwater, especially in regions with specific geological characteristics [80]. Human exposure occurs mainly through contaminated drinking water [81], but also through food consumption [82]. After ingestion, As is efficiently absorbed and undergoes systemic distribution, with the liver acting as the main site of metabolism. Metabolic pathways include reduction and methylation processes leading to urinary excretion, although intermediate species may retain biological reactivity [83].

*Lead (Pb)*

Pb is naturally present in the Earth’s crust, but environmental contamination is largely associated with anthropogenic activities. The use of leaded gasoline has been banned in most countries, and now, major sources of exposure include industrial processes [84]. In the environment, Pb is transported mainly through atmospheric particulate matter and accumulates in soil and water [85]. Human exposure occurs mainly through inhalation of contaminated air and ingestion of food and water, and dust. Additional exposure sources include aging household materials, tobacco smoke, and electronic cigarettes [84,86,87,88,89]. Occupational exposure is relevant in mining, smelting, and battery [84]. Inorganic Pb is distributed throughout the body in a similar manner regardless of the route of exposure, while organic Pb compounds are mainly distributed to the liver, accounting for approximately half of the absorbed dose, with about 5% accumulating in the kidneys and the remainder distributed throughout the body [90].

*Mercury (Hg)*

Hg is present in the Earth’s crust, and it is released into the environment through both natural processes and anthropogenic activities, including industrial emissions, fossil fuel combustion, and waste incineration. It is widely distributed in the air, water, soil, and biota [91]. Hg occurs as elemental, inorganic, and organic forms, which differ in their environmental behavior and toxicokinetic properties [92]. Elemental Hg is volatile and primarily absorbed via inhalation, representing a relevant exposure route in occupational settings and from dental amalgams [93]. Inorganic Hg is commonly found in environmental matrices as salts or mineral forms, mainly deriving from industrial emissions [92]. Organic Hg, and particularly methylmercury, is generated through environmental transformations and represents the main source of human exposure, largely through the consumption of contaminated fish and seafood [94]. Methylmercury is absorbed through the gastrointestinal tract and widely distributed in the body, including the brain (causing neurotoxicity), liver, kidneys, and developing fetus, due to its ability to cross biological barriers such as the blood–brain barrier and placenta [95].

*Chromium (Cr)*

The most toxicologically relevant forms of Cr are trivalent Cr [Cr(III)] and hexavalent Cr [Cr(VI)] [96]. Cr(III) is the predominant natural form and is generally less soluble and less mobile, whereas Cr(VI), mainly generated through industrial activities, is more soluble, mobile, and bioavailable [97]. Environmental Cr originates from both natural sources and anthropogenic activities [96]. Cr is transported as particulate matter and deposited in soil and water. Although it may accumulate in plants, biomagnification through the food chain appears limited [98]. A common source of Cr exposure is food, while occupational exposure is significant in industrial settings. Additional sources include consumer products such as cement, cleaning agents, textiles, leather, and metal prostheses [99]. Absorption depends on the oxidation state, with Cr(VI) being more readily absorbed than Cr(III). Once absorbed, Cr is distributed to multiple tissues and excreted mainly through urine [100].

*Manganese (Mn)*

Mn is a naturally occurring transition metal widely distributed in the Earth’s crust and commonly found in soil, water, and food. Its environmental distribution is influenced by both natural sources and anthropogenic activities [101]. Once released into the environment, Mn can be transported through air, water, and soil [102]. Dietary intake represents the primary source of Mn exposure, as it is naturally present in a wide range of foods and water, particularly in areas with elevated natural Mn levels [103,104]. In contrast, inhalation of airborne Mn is generally limited to specific environmental or occupational settings (mining, welding, steel production, and battery manufacturing) [101]. Following exposure, Mn is absorbed through the gastrointestinal tract and, to a lesser extent, via inhalation.

### 2.2. Endocrine Disruptors: Overview

The “endocrine-disrupting chemical (EDC)” (or endocrine disruptor) is defined as “an exogenous chemical, or mixture of chemicals, that can interfere with any aspect of hormone action” [105]. Interestingly, despite the term “endocrine disruptor” being first introduced in the early ’90 [106], the concept of chemical interference with endocrine function has been recognized for several decades [107].

EDCs can affect multiple aspects of endocrine function, with consequences on growth, development, reproduction, and metabolism [108]. The severity of clinical adverse effects depends above all on the timing, duration, and intensity of exposure. Embryos, fetuses, newborns, and children are more vulnerable to the adverse effects of EDCs due to immature detoxification and metabolic pathways [109]. Maternal exposure during pregnancy and lactation can result in placental and breast milk transfer, with potential long-term effects.

Experimental and epidemiological studies have linked EDC exposure to the risk of endocrine disorders, including hormone-dependent cancers, metabolic diseases, and reproductive dysfunctions [110].

Early-life exposure has been associated with chronic alterations in endocrine, immune, and neurological functions as well as with metabolic dysfunctions, partly mediated by epigenetic mechanisms that may extend across the lifespan [111,112].

In response to accumulating evidence of their potential adverse health effects, EDCs are increasingly subject to regulatory actions, particularly in the European Union and the United States, and in light of their substantial social and economic burden [113].

**Figure 2:**
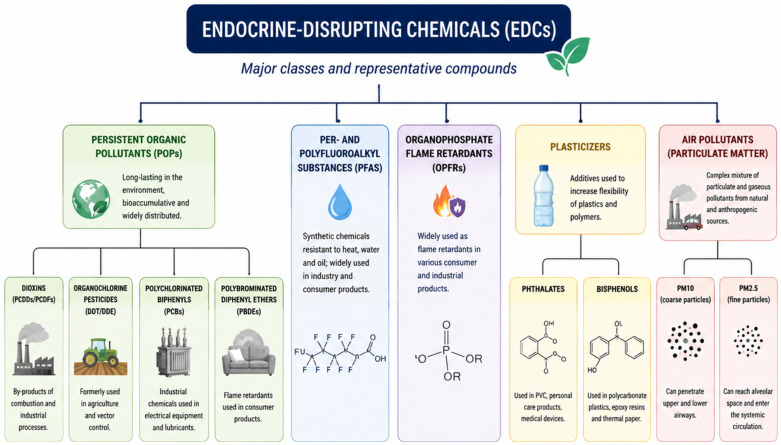
Tree-chart overview of the main classes of endocrine-disrupting chemicals (EDCs) and pollutants discussed in this review. The figure summarizes the major categories, including persistent organic pollutants (POPs), per- and polyfluoroalkyl substances (PFAS), organophosphate flame retardants (OPFRs), plasticizers (phthalates and bisphenols), and air pollutants (particulate matter).

Focusing on EDCs known to interfere with the hypothalamic-pituitary-thyroid (HPT) axis (“thyroid disruptors”), the following sections provide an overview of the main classes and compounds implicated in thyroid hormone synthesis, transport, metabolism, and signaling. A schematic overview of the main categories and representative compounds discussed in this review is provided in Figure 2.

#### 2.2.1. Persistent Organic Pollutants (POPs)

POPs are a group of chemicals that originate from human activities during production, utilization, and disposal. POPs are characterized by high chemical stability, with the peculiarity of having long half-lives in soils (generally years), sediments, air (several days), or biota. Considering their affinity for lipids, POPs accumulate in adipose tissue, resulting in very high persistence and chronic exposure at very low concentrations. These compounds are capable of long-range environmental transport through air and water. This can cause contamination of regions far from their original source, especially via the food chain [113].

The Stockholm Convention on POPs, together with its subsequent amendments, has identified and regulated several classes of nonpolar organic chemicals as POPs, including organochlorine pesticides, polychlorinated biphenyls (PCBs), and polychlorinated dibenzo-p-dioxins (PCDs) (Table 2) [114].

*Polychlorinated Biphenyls (PCBs) and Polybrominated diphenyl ethers (PBDEs)*

PCBs and PBDEs represent the two major classes of POPs, although other brominated compounds, such as polybrominated biphenyls (PBBs) are recognized as legacy environmental contaminants. Both groups are characterized by high chemical stability, lipophilicity, and resistance to thermal degradation [115]. Although their production has been banned in many countries, both PCBs and PBDEs still represent ubiquitous environmental contaminants due to their long half-life and bioaccumulative properties [115]. Human exposure occurs mainly via dietary intake [116]. These compounds accumulate in adipose tissue and are detectable in blood, breast milk, and placental and fetal tissue. Additional exposure may occur through inhalation [117].

*Dioxins*

Dioxins, including polychlorinated dibenzo-p-dioxins (PCDDs) and dibenzofurans (PCDFs), are highly toxic POPs generated mainly by combustion processes and industrial activities, as well as from natural events, including volcanic eruptions and lightning disasters [118]. Their planar aromatic structure confers environmental persistence and bioaccumulation [119]. The history of dioxin exposure is a 200-year timeline of industrial accidents, chemical warfare, and environmental disasters. In particular, in 1976, an industrial explosion in Seveso, Italy, released a toxic cloud with TCDD concentrations reaching unprecedented levels (up to 100 ppm). Studies from exposed people confirmed that dioxins act as potent endocrine disruptors, altering thyroid and reproductive functions, increasing cardiovascular risk, and serving as a multi-organ cancer trigger [120]. Human exposure occurs mainly through contaminated food. Due to its bioaccumulative properties, dioxin persists for long periods in the body and can be transferred prenatally and through breastfeeding. [121].

*Organochlorine pesticides*

Organochlorine pesticides (OCPs), including DDT, are persistent synthetic compounds formerly used in agriculture and vector control [122] and still detectable in the environment despite regulatory restrictions [123]. Chemically, OCPs are composed of aromatic rings heavily substituted with chlorine atoms. Their marked chemical stability and lipophilicity make these compounds resistant to photolysis and degradation [124].

OCPs bioaccumulate in the food chain, and human exposure occurs mainly through contaminated food, particularly animal-derived products [125]. Maternal exposure can result in placental transfer during pregnancy and excretion through breast milk, thereby contributing to prenatal and early-life exposure [126].

*Per- and polyfluoroalkyl substances (PFAS)*

PFAS represent a class of synthetic fluorinated compounds characterized by exceptional chemical stability and environmental persistence, earning the designation “forever chemicals” [127]. The most extensively studied PFAS are perfluorooctane sulfonate (PFOS) and perfluorooctanoic acid (PFOA), used in industrial and commercial applications, and listed as POPs under the Stockholm Convention. PFAS have been widely employed in industrial and consumer products [128]. Human exposure occurs mainly through contaminated food and water [129].

Unlike classical lipophilic POPs, PFAS do not preferentially accumulate in adipose tissue, but they bind to circulating serum proteins, particularly albumin, and distribute predominantly in blood, liver, and kidneys [130].

Despite regulatory restrictions, their environmental persistence and long biological half-lives result in sustained internal exposure even after their production declines.

#### 2.2.2. Flame Retardants Non-PBDE

Organophosphate flame retardants (OPFRs) are synthetic organophosphorus compounds used in place of PBDEs, following progressive regulatory restrictions on brominated flame retardants [131]. Although less persistent than classical POPs, OPFRs are characterized by greater chemical mobility, and they easily migrate from treated materials into surrounding environments [132]. These compounds are commonly found in furniture, textiles, electronic devices, and construction materials, with indoor dust and air representing the main exposure routes [133]. Ingestion of contaminated indoor dust represents a significant route for human exposure [133]. Once absorbed, OPFRs are distributed in various tissues, but generally metabolized and excreted relatively rapidly compared to more persistent POPs.

#### 2.2.3. Plasticizers and Phenolic Compounds

*Phthalate*

Phthalates, a group of chemical compounds deriving from phthalic acid, are produced as diesters, such as diethylhexyl phthalate (DEHP), dibutyl phthalate (DBP), and diethyl phthalate (DEP) [134]. Phthalates are widely used plasticizers found in numerous consumer products, including food packaging, personal care products, and medical devices [135]. Human exposure occurs mainly through dietary intake [134], but also via inhalation, dermal absorption, and medical devices [136].

After exposure, phthalates are rapidly metabolized into monoesters and oxidative metabolites. Multiple lines of evidence confirmed a widespread human contamination of phthalates and their metabolites, showing high distribution in urine, blood, hair, amniotic fluid, and breast milk [137].

*Bisphenols*

Bisphenols are a class of synthetic organic compounds characterized by two phenolic rings connected by a bridging group [138]. Among them, bisphenol A (BPA) is the most extensively studied. It is widely adopted for industrial applications and therefore incorporated into plastics, epoxy resins, food-contact materials, thermal paper, and other consumer products [139]. Human exposure occurs mainly through dietary intake [140], as BPA can migrate into food, particularly under conditions of heat, repeated use, or prolonged storage, although dermal absorption and inhalation may also contribute.

### 2.3. Environmental Air Pollutants with Endocrine-Disrupting Potential

Ambient air pollution is represented by a complex and dynamic mixture of gaseous compounds and particulate contaminants. Among these, particulate matter (PM), classified into PM_10_ and PM_2.5_ according to aerodynamic diameter, is a major environmental risk factor originating from both natural and anthropogenic sources, particularly traffic and industrial emissions [141]. PM represents a complex surface matrix capable of adsorbing and transporting a wide range of toxic chemicals, including HMs, polycyclic aromatic hydrocarbons, volatile organic compounds, and other substances with recognized endocrine-disrupting potential. Therefore, PM acts not only as a direct toxic agent but also as a “carrier” of other endocrine-active pollutants [142].

Due to their small size, PM_10_ particles can penetrate the upper and lower airways, whereas PM_2_._5_ particles can reach the alveolar space and, in part, translocate into the systemic circulation. Although studied in relation to respiratory and cardiovascular outcomes, growing evidence indicates that chronic exposure may also affect endocrine and metabolic regulation and acts as a thyroid disruptor [143].

## 3. Environmental Exposure and Non-Neoplastic Thyroid Diseases

Exposure to environmental pollutants has been linked to a broad spectrum of diseases, including respiratory diseases, neurodevelopmental disorders, cardio-metabolic diseases, reproductive abnormalities, immune alteration, as well as neoplastic diseases [144]. Notably, there is growing evidence that HMs and classical EDCs can interfere with hormonal synthesis and regulation, inducing alterations in HPT axis activity and negative feedback regulation, inhibition of iodine uptake, and interference with thyroid hormone synthesis or metabolism [145,146]. These effects contribute to the development of hypothyroidism, hyperthyroidism, or autoimmune thyroid diseases. Epidemiological studies have identified significant correlations between exposure to POPs, HMs, and air pollutants, with changes in thyroid hormone parameters as well as with other non-malignant thyroid diseases, such as thyroid nodules [147]. However, findings are sometimes challenging to reconcile, considering discrepancies in exposure measurement, population characteristics, and clinical outcome definitions. Furthermore, many studies have focused on isolated pollutants, overlooking the synergistic effects inherent in real-world co-exposure of different chemicals.

### 3.1. Heavy Metals and Thyroid Dysfunctions

The thyroid gland is the most sensitive component of the endocrine system regarding environmental insults. Its unique characteristic is the physiological requirement for iodine, which makes the thyroid gland functionally vulnerable to “molecular mimicry” by HMs. Furthermore, the active uptake of toxic metals that share chemical similarities with essential trace elements is promoted by their high vascularization [148]. Although the relationship between HM exposure is less studied for non-neoplastic thyroid diseases compared with TC, several studies have suggested that exposure to HMs may lead to conditions such as hypothyroidism, hyperthyroidism, and thyroid autoimmunity [149]. The individual susceptibilities, such as genetic predisposition and nutritional status, including iodine intake, also reinforce the complexity of these associations, making epidemiological studies further challenging [150]. In addition, unlike organic pollutants, HMs are persistent, non-biodegradable, and bioaccumulative, often reaching concentrations in the thyroid parenchyma that far exceed those found in the blood. From both a clinical and pathophysiological point of view, the impact of HMs on the thyroid extends beyond their direct toxicity and has to be considered within an immunological framework. In this setting, increasing evidence suggests that HMs may act as environmental “triggers” that precipitate thyroid autoimmunity in genetically predisposed individuals [149]. Chronic low-dose exposure to metals such as Cd, Hg, and Pb promotes persistent oxidative stress within the thyrocyte [151,152]. Pamphlett et al. detected intracellular HMs such as Hg, Cd, Pb, Ni, and silver in follicular cells of human subjects, with prevalence for Hg, increasing with age from 4% in individuals <30 years to 38% in those over 60 years [151]. The thyroid gland is particularly vulnerable due to continuous exposure to hydrogen peroxide generated during hormone synthesis. In this pro-oxidant microenvironment, the excessive accumulation of ROS triggers inflammation and damage, which may increase antigenicity, facilitating loss of immune tolerance and progressive lymphocytic infiltration [153]. Epidemiological data summarized in recent systematic reviews support this model, demonstrating associations between heavy metal exposure and altered TSH, FT4, and FT3 levels, as well as increased prevalence of thyroid autoimmunity and non-malignant structural thyroid abnormalities [147,149]. Beyond autoimmunity, HMs can disrupt thyroid function at multiple levels of the HPT axis, as well as at peripheral levels on thyroid hormone receptor activities [56] (see Section 5).

*Cadmium (Cd)*

The association between Cd exposure and thyroid disorders has been confirmed by several studies and meta-analyses [154]. Cd acts as an endocrine disruptor by impairing iodide uptake, inhibiting TPO activity, altering peripheral thyroid hormone metabolism, and disrupting the HPT axis. It competes with Se-dependent enzymes, reducing deiodinase activity and leading to relatively reduced FT3 levels despite normal TSH levels [147]. Patients may present with nonspecific hypothyroid symptoms (fatigue, weight gain, cold intolerance) with borderline laboratory findings. Several retrospective studies have linked Cd exposure to alterations in thyroid function [53,155]. Analyses of NHANES 2007–2008 data showed that higher urinary Cd levels were associated with increased thyroid hormones and Tg levels, while TSH was inconsistently related to Cd exposure [53]. Other analyses of the same dataset reported positive associations between urinary Cd and T3/T4 and an inverse relationship between blood Cd and TSH, suggesting a possible link between Cd exposure and thyroid dysfunction [156]. Overall, thyroid hormone levels may reflect cumulative Cd burden, while the effect on TSH may indicate more recent exposure [62]. Occupational studies have also linked increased Cd exposure to altered thyroid function, particularly elevated TSH levels [157], but data are conflicting, and other authors found no significant associations [158]. More recent population studies suggest that Cd may also contribute to thyroid autoimmunity, thyroid structure changes, and hypothyroid status, particularly in women [159,160]. However, human evidence on Cd effects on thyroid function remains debated, reflecting differences in exposure assessment, study populations, and duration or route of Cd exposure [62].

*Mercury (Hg)*

Hg exposure has been linked to autoimmune thyroid disease. Epidemiological studies indicate associations between higher Hg levels and increased prevalence of thyroid autoantibodies [147]. The analysis performed on NHANES 2007–2008 data in 2047 adult women found that higher blood Hg levels were associated with increased odds of thyroglobulin antibody positivity, despite no association being observed for anti-TPO autoantibodies (TPOAb) [47]. Concerning thyroid function, although Chen et al. (NHANES 2007–2008 data) reported no significant association between Hg exposure and thyroid hormone levels, a meta-analysis including 13 studies reported that Hg exposure in the general population was associated with alterations in thyroid hormones, particularly increased TSH and FT4 levels, with variable effects on T4 [161]. Furthermore, even at low exposure levels, inorganic Hg has been associated with reduced maternal T3 and lower cord serum FT4 during pregnancy, suggesting its role in inducing a subtle thyroid disruption during a critical window for fetal neurodevelopment [162].

*Lead (Pb)*

Pb remains a pervasive HM that affects the thyroid through a particular mechanism. While the other above-mentioned metals target the gland directly, Pb primarily disrupts the central regulation of the HPT axis, leading not only to reduced TSH levels, but also GH and gonadotropin in response to their releasing factors (TRH, GHRH, and GnRH, respectively) [163]. Consequently, Pb-exposed individuals may suffer from “secondary” or “central” hypothyroidism, according to findings that showed decreased total T4 concentration after Pb exposure, without the expected “appropriate” significant association with increased TSH levels [164]. Potential direct effects of Pb on thyroid function have been reported, suggesting that Pb exposure affects both peripheral thyroid hormone levels and TSH. In one of the first studies, Slingerland demonstrated reduced iodine uptake by the thyroid gland in cases of high Pb exposure [165], according to both rat models and in vivo studies [166]. Chronic exposure has also been associated with structural changes in the thyroid gland, including a reduction in thyroid follicle size and nuclear alterations in follicular cells [167]. Most clinical studies have linked Pb exposure to thyroid dysfunctions and hypothyroidism [168], particularly in individuals with occupational exposure to this metal [169]. A recent Italian study involving workers from a petrochemical company exposed to inorganic Pb compounds demonstrated that higher blood Pb levels were associated with reduced TSH and increased FT3 and FT4 concentrations, suggesting a trend toward hyperthyroidism [170]. Furthermore, greater exposure was associated with higher TPOAb levels, suggesting a possible link between Pb exposure and increased susceptibility to thyroid autoimmunity [170].

*Other metals*

Evidence on the effects of other metals, such as Mn, Cu, As, and Cr, on thyroid function is more limited and heterogeneous. Epidemiological studies have suggested that environmental exposure to Mn may be associated with subtle alterations in circulating FT4 and TSH levels [158], potentially inducing both hypothyroidism and hyperthyroidism, with possible thyroid function imbalance leading to adverse neurodevelopmental outcomes [171]. Exposure, particularly through contaminated drinking water, has been linked in some human studies with reduced circulating thyroid hormone levels, increased TSH concentrations [172], and a higher prevalence of goiter [173]. Cu concentration has been reported to be altered in some thyroid disorders [174] and thyroid nodules [175], although a causal relationship with thyroid dysfunction remains unclear. Similarly, evidence regarding Cr and thyroid function remains inconsistent. While a few studies reported significant associations with TSH and FT4 levels, the majority of available studies found no significant relationship [149]. Therefore, current data suggest that these metals may influence thyroid homeostasis, but their clinical relevance in non-malignant thyroid disease remains a matter of debate and requires further investigation.

### 3.2. EDCs and Thyroid Dysfunctions

EDCs represent one of the most extensively studied groups of compounds able to interfere with thyroid homeostasis. The HPT axis is particularly vulnerable to endocrine disruption because of its effects on thyroid hormone synthesis, transport, metabolism, and receptor signaling. However, in contrast to HMs, which often exert direct toxic effects on thyrocytes or immune responses, many EDCs disrupt thyroid function through molecular mimicry or interference with hormone transport [176]. Therefore, EDCs’ toxic effects involve multiple biochemical steps. Epidemiological studies have reported associations between exposure to several EDC classes and alterations in thyroid function, as well as with an increased prevalence of autoimmune thyroid disease and structural thyroid abnormalities [177]. However, the interpretation of these associations remains challenging based on differences in exposure assessment, inter-individual susceptibility, and the complex mixture of pollutants encountered in real-world environmental exposure scenarios.

*POPs and OPFRs*

POPs are among the best studied thyroid-disruptors in humans. Within POPs, PCBs and PBDEs deserve particular attention because of their structural similarity to thyroid hormones. An important contribution in this field comes from the Michigan PBB Registry, established after a PBB food supply contamination occurred in 1973–1974. Jacobson et al. found an association between PBB and PCB contamination and thyroid diseases (hypothyroidism, hyperthyroidism, thyroid nodule, and thyroiditis), suggesting that susceptibility may be greater in selected subgroups, including women with low iodine status [178]. In the same registry, Curtis reported that PBB and PCB exposure was associated with thyroid hormone (but not TSH) alterations, with stronger effects in subjects exposed during childhood or prenatally, suggesting a possible “endocrine footprint” of these compounds that may persist for decades after exposure [179]. Further evidence comes from human populations with chronic exposure to PCB mixtures. In the Great Lakes population, Turyk et al. reported significant negative associations between PCBs and total T3, total T4, and TSH [180]. In eastern Slovakia, a heavily contaminated area, Langer and colleagues described increased thyroid volume and higher prevalence of TPOAb and anti-TSH receptor antibodies (TRAb) in a cohort of young adults (21–35 years) from an organochlorines-polluted area compared to an age-matched cohort from a background pollution area, suggesting a link between organochlorine exposure and thyroid dysfunction [181]. However, not all high-exposure cohorts have shown a clear association with thyroid function, underscoring how the thyroid-disrupting potential of POPs may depend on several variables, including age at exposure, gender, iodine intake, and genetic background [182]. Pregnancy and early life represent the clinical setting in which the evidence for thyroid-disrupting effects of POPs is probably most compelling. Several mother–child cohort studies have documented associations between prenatal PCB, PBDE, DDE, PFOS, or PFOA exposure and neonatal thyroid hormone alterations. In a Dutch prospective cohort study, exposure to DDE, PFOS, and PFOA was associated with newborn T4 concentrations in a sex-specific manner (increased T4 in girls in the highest quartile of DDE and PFOA exposure and lower T4 in boys in the second quartile of PFOS and PFOA exposure [183]). Moreover, a possible negative effect of perinatal exposure to PCBs on deiodinase type 3 activity has been observed, suggesting disruption not only of hormone synthesis but also of peripheral thyroid hormone metabolism [184]. Concerning PBDEs, some findings suggest a positive association between maternal exposure, particularly to BDE-28 and BDE-47, and maternal T4 and T3 concentrations during pregnancy [185], and an inverse association between prenatal exposure to low levels of PBDE and thyroid hormones in cord plasma has been proposed [186]. Altogether, these studies support the concept that even background environmental exposure may perturb maternal-fetal thyroid homeostasis.

OCPs also appear clinically relevant with human studies from highly polluted areas, suggesting that mixtures containing DDE, HCB, and related organochlorines may associate not only with biochemical thyroid alterations but also with sonographic thyroid enlargement and autoimmune markers [187].

PFAS are increasingly recognized as thyroid disruptors in human studies. An epidemiological analysis based on NHANES data showed that higher serum PFOA and PFOS concentrations were associated with thyroid disease in the U.S. adult population, especially in women [188]. In line with this, different studies suggested a possible interference of PFOA and PFOS with thyroid peripheral metabolism, transport, and cellular uptake [189] and an association in pregnancy cohorts between PFAS exposure and maternal or neonatal thyroid hormones, although the direction of the associations was not always uniform [190,191]. Overall, these results are particularly significant because they show that clinically relevant thyroid disruption may occur at exposure levels comparable to those of the general population and not only in extreme contamination scenarios. A recent study examined the impact of the combined exposure to nine PFAS and heavy metals mixtures on thyroid function in pregnant women of different age groups. The authors observed a negative association between the above-mentioned co-exposure and FT3 levels, especially in the non-advanced age group [192]. A more detailed analysis identified PFOA as a primary contributor to thyroid dysfunction. Interestingly, this effect seemed to be mitigated by some hematological parameters. Specifically, the authors found that hemoglobin levels masked approximately 15% of the negative correlation between chemical co-exposure and thyroid hormones [192]. Therefore, it has been hypothesized that pollutants may exert their thyrotoxic effects not only through direct glandular interference but also indirectly by altering erythropoiesis and heme synthesis, identifying hemoglobin as a potential physiological mediator that could be targeted to mitigate environmental endocrine disruption [192]. Compared with classical POPs, evidence on OPFRs is more recent but increasingly concerning. Indeed, these modern flame retardants are not biologically inert, with growing evidence showing thyroid-disrupting potential, especially in pregnant women and newborns [193,194].

*Plasticizers*

A meta-analysis including 13 studies suggested a significant association between exposure to DEHP and HPT axis regulation. In particular, the urinary concentration of two DEHP metabolites was negatively correlated with total T4 levels, and the concentration of another metabolite was positively correlated with TSH levels [195]. Both longitudinal studies and cohort studies observed a dose-dependent disruption of thyroid function by phthalate mixtures, mainly characterized by reduced T4/FT4 and increased TSH, with particularly relevant effects during pregnancy [196]. These studies are particularly relevant because they move beyond single-compound analyses and address repeated measures and mixture effects, which better reflect clinical reality. BPA effects on thyroid function are likewise supported by several human studies. A recent systematic review with meta-analysis showed that the relationship between BPA and thyroid function can be different according to gender, with a positive correlation with FT4 in females and a negative correlation with FT4 and TSH in males [197]. Similarly to the previously discussed EDCs, also for BPA and its derivatives, studies support more important effects on thyroid function for exposure during pregnancy [198]. Finally, some studies investigated possible effects on structural thyroid disease, but without conclusive findings [183]. Increasing attention has been directed toward PFAS, commonly associated with non-stick cookware, food packaging materials, and contaminated drinking water, and in particular PFOA, which has been associated with thyroid disease [188]. Epidemiological studies have reported modest increases in TSH levels and, less consistently, reductions in FT4 concentrations, suggesting a shift toward a hypothyroid-like profile or a dysregulation of the HPT axis, more evident in women and during pregnancy.

Taken together, current clinical evidence indicates that multiple EDC classes can interfere with thyroid function in humans, with the strongest data deriving from POPs, PFAS, phthalates, and BPA. The most relevant findings include altered TSH and FT4 concentrations and disruption of maternal-fetal thyroid homeostasis. However, as already observed for HMs, inconsistencies across studies remain frequent and probably reflect the combined effects of multiple variables, including sex, age, pregnancy, iodine status, exposure timing, and pollutant mixtures. Overall, EDC-related thyroid dysfunction should not be interpreted as the consequence of a single compound but rather as the result of chronic cumulative exposure, probably acting on a biologically vulnerable endocrine axis. Future longitudinal studies, including demographic, clinical, and environmental variables, with repeated thyroid function tests and imaging assessment, will be essential to better define the clinical relevance of these associations and to identify the subgroups most susceptible to environmental thyroid disruption.

## 4. Thyroid Carcinogenesis: Role of HMs and EDCs

Epidemiological research investigating the relationship between HMs, EDCs, and TC has expanded considerably in recent years. However, the available evidence remains heterogeneous in both strength and consistency across pollutant classes. To provide a structured and integrated overview, this section presents a complementary perspective on exposure characteristics, mechanistic insights, and quantitative evidence synthesis. It also examines current epidemiological data by pollutant class, categorized into seven major groups of environmental contaminants, including HMs, PFAS, OCPs, POPs, OPFRs, plasticizers and bisphenols, as well as other environmental contaminants such as nitrate. The information summarized in Table 3 highlights the widespread distribution of these pollutants across environmental compartments, including air, water, soil, and food, and provides the environmental and exposure context necessary to interpret epidemiological findings. Regulatory status varies across pollutant classes, ranging from complete bans (e.g., DDT, PCBs in many countries) to partial restrictions or ongoing regulation (e.g., PFAS, bisphenols, and phthalates).

### 4.1. HMs and TC Risk

Many studies have investigated the possible association between HM exposure and thyroid carcinogenesis, although the specific mechanisms underlying this relationship remain incompletely understood. Metals such as Cd, As, Ni, and Cr have been classified by the International Agency for Research on Cancer (WHO–IARC) as carcinogenic to humans, placing them in Groups 1 and 2 (monograph.iarc.who.int/list of classifications accessed on 25 April 2026). Table 4 summarizes the main experimental evidence on thyroid-related effects induced by HMs and EDCs, providing a structured overview of observed alterations across different models. This table is intended as a descriptive synthesis, while the underlying molecular mechanisms and their integration into thyroid pathophysiology are discussed in detail in the following section.

Various mechanisms related to HM exposure have been found to play a carcinogenic role, including oxidative stress, DNA damage, epigenetic alterations, and endocrine interference. The carcinogenic effect of HMs on target cells depends on several biological factors, such as bioavailability, the intracellular distribution, and interactions with cellular proteins and enzymes. These steps are tissue and cell-specific [216]. Epidemiological studies reported that TC incidence is particularly increased in geographical regions characterized by high levels of HM contamination, and a cause-effect relationship between metal exposure and thyroid carcinogenesis has been hypothesized. Many studies have shown that the thyroid gland can accumulate various types of HMs, impacting thyroid function and homeostasis through the inactivation of antioxidant defense mechanisms, primarily through their interaction with intracellular glutathione (GSH) or sulfhydryl groups (R-SH) of antioxidant enzymes such as superoxide dismutase (SOD), catalase, glutathione peroxidase (GPx), and glutathione reductase (GR) [217]. In particular, in vitro and in vivo experiments support the hypothesis that chronic exposure to HMs at slightly increased environmental concentrations may predispose thyroid stem/progenitor cells to malignant transformation. The threshold and dose–response relationship differ substantially across metals. For essential metals, protective effects may operate only with narrow physiological ranges, above which toxicity emerges [218]. In vitro experiments with thyroid stem/progenitor cells revealed a biphasic dose–response curve for metal exposure (hormesis effect). This hormesis pattern may explain why small environmental increases can alter stem cell biology and predispose to malignant transformation, particularly when exposure is chronic rather than acute [219]. Many clinical observations suggest a possible carcinogenic role for HMs in thyroid tissue, similar to other EDCs. Indeed, it is well known that an increased incidence of TC was reported in volcanic areas around the world [33,217,220]. Furthermore, a higher excess risk of TC was also documented in areas and regions characterized by intensive industrial activities and in those with intensive mining activity [10,216,220,221,222]. The HMs most frequently studied and potentially associated with thyroid carcinogenesis are Cd, Hg, and Mn, although their role in thyroid carcinogenesis has not been established. The above-mentioned evidence underscores the multifactorial nature of environmental influences on thyroid carcinogenesis.

*Manganese (Mn)*

Van Gerwen et al., in their meta-analysis including 19 studies with a total of 6095 patients, reported a significant positive association between Mn tissue levels and TC and a significant negative association with cobalt blood levels, suggesting that the bioaccumulation in thyroid tissue of this element may drive carcinogenic effects [210]. Another study, performing a two-sample Mendelian randomization (MR) analysis to assess the causal relationships between 21 metal elements and TC risk in East Asian populations, confirmed evidence for causal links between elevated Mn and Pb levels and the risk of TC [223].

*Mercury (Hg)*

The carcinogenic role of Hg in thyroid tumors is not very clear, although some evidence suggests a potential role of this metal in the onset and/or development of TC. A recent review, reporting nine studies performed between 1995 and 2022, highlighted a probable association between TC risk and Hg exposure. Patients affected by TC presented significantly higher Hg levels, both in thyroid tissues than in urine samples, compared to those with normal thyroid, substantiating Hg as a crucial contributor to thyroid carcinogenesis. However, limitations on the study designs do not allow for assessing a dose–response relationship between Hg and TC [211]. Other Authors reported a significant dose–response relationship between Hg levels and the risk of PTC [224]. A cross-sectional study, based on a large database analysis, investigated the association between TC incidence and three metals (Pb, Cd, and Hg) with increased levels in soil samples in Puerto Rico, reporting a negative correlation between Hg exposure and TC [225]. Interestingly, Kim et al. documented that urinary Hg concentration was positively associated with the risk of TC among residents living near national industrial complexes, suggesting the possible hypothesis that environmental HM pollution may contribute to the development of TC in the South Korean population [48].

*Cadmium (Cd)*

Cd is known to act as a thyroid disruptor and carcinogen in humans, although its role in TC is unclear. A prospective study population conducted in South Korea, evaluating the associations between pre-diagnostic urinary Cd concentrations and TC risk, suggested that low-level environmental exposure to Cd may increase TC risk [212]. Some authors evaluating the presence of HMs in neoplastic tissue of 66 PTCs and the corresponding healthy contralateral tissues of the same patients reported that Cd acts as the main endocrine disruptor [226]. Other authors identified the chronic Cd accumulation in thyroid tissue as one of the aggravating factors for TC progression [227]. While most studies focus on the effect of exposure to individual metal ions, examining exposure to mixtures of metal ions may offer a more accurate reflection of environmental exposure. Some authors reported in their meta-analysis, using a random effect model, a positive correlation between heavy metals and TC risk (Standardized mean difference (SMD) = 0.21, 95%CI = (0.11, 0.32), *p* < 0.001). Particularly, the results showed that Cd, Pb, Hg, and As were positively associated with TC risk, while the association between Cr and Cu indicated no statistical significance [63]. A case–control study conducted in China reported a significant association between urinary concentrations of several metals and PTC [224]. A recent study reported that benign and malignant thyroid tissues exhibit different trace-element profiles. In particular the malignant group exhibited significantly higher concentrations in eight elements (magnesium (Mg), aluminum (Al), Fe, Cr, titanium (Ti), strontium (Sr), tin (Sn), barium (Ba)] compared to the benign group, while levels of six elements [sodium (Na), Mn, Cu, Zn, Cd, and molybdenum (Mo)) were significantly lower [228].

*Possible association between exposure and more aggressive histological patterns*

To date, we only have some evidence on the relationship between HM exposure and more aggressive histological patterns. The demonstration of a causal link between exposure to a carcinogenic metal and TC phenotype is therefore weak and varies by metal type. Table 5 reports the more representative studies that have investigated this type of relationship and which vary in design, including primary research studies, reviews, and meta-analyses.

Chung et al. [227] examined 92 Korean women undergoing thyroidectomy to evaluate the association between blood and tissue levels of heavy metals and different stages of TC. The study showed that the higher tissue Cd levels were associated with a more advanced stage of TC. Logistic regression analysis revealed the odds ratio for advanced tumor stage (>T2) increased with higher tissue Cd levels (OR = 1.397, 95% CI = 1078–1811). This finding is of particular significance, given that traditional Korean cuisine is linked to high levels of Cd exposure [231].

Ming-jun Hu et al. [229] reported that high Mn levels were associated with increased risk of capsular invasion and advanced T stage (T3/T4a-b), while high cobalt levels showed decreased risk of capsular invasion.

Deng et al. [55] in their meta-analysis reported that Cr (VI) exposure among workers is related to a high risk of death owing to lung, larynx, bladder, kidney, testicular, bone, and TC. In particular, for TC (3 studies, 19,109 cases, 8 deaths), the combined standardized mortality ratio (SMR) was 2.41 (95% CI: 1.19–4.87) [55].

Capezzone et al. [230] reported the number of cases and European age-standardized incidence and mortality rates of TC patients in the Tuscany region (Italy) correlated with the histological records of TC patients. When evaluating the relative risk (RR) of diagnosing TC in relation to environmental sources of HM pollution exposure, the authors found that exposed patients had a RR of 1.16 (95% CI: 1.04–1.29), significantly greater compared to non-exposed patients. Compared to tumors from unexposed patients, TCs from exposed patients were characterized by a lower number of microcarcinomas (*p* = 0.02), a higher rate of extrathyroidal invasion (*p* < 0.001), a higher rate of multicentricity (*p* = 0.002), and bilaterality (*p* < 0.001). The authors concluded that in some geographical areas, the presence of environmental pollution, especially that characterized by the release of HMs, might influence thyroid carcinogenesis and should be considered among the recognized risk factors for TC [230].

### 4.2. EDCs, Pollutants, and TC

The following sections summarize epidemiological associations across EDC classes and, with studies stratified by design (meta-analyses, systematic reviews, and case–control studies), enable a systematic assessment of the level, quality, and consistency of the available evidence and facilitate comparison across pollutant classes.

#### 4.2.1. POPs

POPs, including PCBs and PBDEs, represent one of the most extensively studied classes of EDCs in relation to TC. Evidence from a recent meta-analysis by Yang et al. [63] indicates a positive association between PBDE exposure and TC, whereas findings for PCBs remain inconsistent or suggest inverse associations in some analyses. Prospective evidence is limited. A nested case–control study within the PLCO (Prostate, Lung, Colorectal, and Ovarian) cohort did not identify a significant association between serum PBDE levels and thyroid cancer risk [232]. In contrast, case–control studies have reported positive associations between circulating PBDE congeners and TC, as shown in a Chinese population [206]. Overall, the epidemiological evidence for POPs remains heterogeneous, with more consistent signals for PBDEs than for PCBs.

*Dioxin*

Epidemiological evidence linking dioxins to TC is limited and largely indirect. Most available data derive from occupational or environmentally exposed cohorts, with few studies specifically addressing TC outcomes. Reviews of EDCs suggest that dioxin-like compounds may influence thyroid carcinogenesis; however, direct epidemiological confirmation remains scarce [233].

*OCPs*

OCPs represent one of the pollutant classes with the most consistent epidemiological support. Meta-analyses by Han et al. [204] and Yang et al. [201] reported positive associations between pesticide exposure and TC, particularly for agricultural exposure and specific pesticide subclasses. Cohort and nested case–control studies provide additional, though more variable, evidence. For example, Lerro et al. [234] identified compound-specific associations rather than uniform effects across all pesticides. Case–control studies have also consistently reported associations with DDT/DDE and related compounds. Taken together, pesticides show the most robust and consistent epidemiological evidence among environmental pollutants.

*PFAS (PFOA, PFOS)*

The epidemiological evidence for PFAS remains limited and inconsistent. Recent systematic reviews and meta-analyses have not identified statistically significant overall associations between PFAS exposure and TC [200,235]. However, some case–control studies suggest compound-specific associations. For instance, Cirello et al. [236] reported associations between selected PFAS and thyroid cancer or thyroid-related conditions, although findings were not consistent across compounds. Thus, while mechanistic plausibility is strong, epidemiological evidence remains inconclusive.

#### 4.2.2. Non-PBDE Flame Retardants

For newer flame retardants, including OPFRs, epidemiological data are still scarce. Current evidence is largely derived from biomonitoring studies and broader reviews of EDCs, with limited direct evaluation of TC risk [233]. This class remains an emerging area of research.

#### 4.2.3. Plasticizers and Phenolic Compounds

Phthalates have been increasingly investigated in recent years. The meta-analysis by Yang et al. [63] reported a positive association between phthalate exposure and TC. Supporting these findings, Liu et al. [207] observed that urinary metabolites of phthalates were associated with increased risks of TC and benign thyroid nodules. Although most studies rely on case–control designs and biomonitoring data, the consistency of findings suggests moderate epidemiological evidence. Bisphenols have been investigated primarily in case–control studies. Zhang et al. [208] reported associations between urinary bisphenol levels and PTC. However, the overall evidence remains limited and is largely supported by mechanistic data rather than robust epidemiological studies. Table 6 presents the main meta-analyses investigating the association between environmental pollutants and thyroid cancer or thyroid-related outcomes, updated through 2025. For each study, the table reports authors, year of publication, pollutant class, compounds evaluated, outcome assessed, and the principal findings. The inclusion of both TC and thyroid-related outcomes reflects the current state of the literature, where epidemiological evidence often derives not only from studies directly assessing cancer risk, but also from investigations of intermediate endpoints such as thyroid dysfunction and nodular disease. These outcomes may represent early biological effects of environmental exposures and provide insight into potential pathways leading to carcinogenesis. Overall, the meta-analyses summarized reveal a heterogeneous pattern of associations across pollutant classes. More consistent positive associations are observed for pesticide exposure and, to a lesser extent, for certain heavy metals and phthalates, whereas PFAS and other EDCs show limited or inconsistent pooled results. The table also highlights important methodological limitations, including reliance on observational data, variability in exposure assessment, and heterogeneity across studies.

### 4.3. Early Life Exposure

Evidence directly linking early-life exposure to environmental pollutants and TC remains limited and should be interpreted with caution. To date, the most consistent and well-established evidence supporting an early window of susceptibility relates to exposure to ionizing radiation during childhood [237,238] (Table 7).

In contrast, epidemiological data directly linking prenatal or early-life exposure to EDCs and other environmental pollutants with TC risk are still scarce. Nevertheless, several lines of evidence support the biological plausibility of an early-life susceptibility contribution (Table 7). Large population-based studies have identified associations between TC risk and perinatal factors, including higher birth weight, congenital hypothyroidism, and maternal metabolic or thyroid conditions during pregnancy [239,240]. In addition, pediatric and adolescent data further support the relevance of early-life exposures; for example, a population-based study of childhood TC in California highlighted the potential contribution of birth characteristics and early-life factors to disease risk [241]. These findings suggest that exposures occurring during critical developmental windows may influence disease risk later in life. In parallel, experimental and human studies have demonstrated that various EDCs—including PFAS, pesticides, and POPs—can interfere with thyroid hormone homeostasis, particularly during fetal and early postnatal development [233,242,243,244] (Table 7). Although most epidemiological studies on these compounds have focused on adult exposures, their well-documented effects on thyroid function during early life support a potential role in long-term carcinogenic processes. Recent epidemiological evidence also suggests a possible contribution of prenatal exposure to environmental contaminants. A population-based study conducted in California reported associations between prenatal exposure to PFAS from contaminated drinking water and increased risk of childhood cancers [246]. While not specific to TC, these findings reinforce the concept that early-life exposure to persistent pollutants may influence cancer susceptibility. Overall, while direct epidemiological evidence remains limited, the combination of mechanistic data and indirect epidemiological findings supports the hypothesis that early-life exposure to environmental pollutants may contribute to thyroid carcinogenesis. Future research should prioritize prospective cohort studies with exposure assessment during pregnancy and early life.

**Figure 3:**
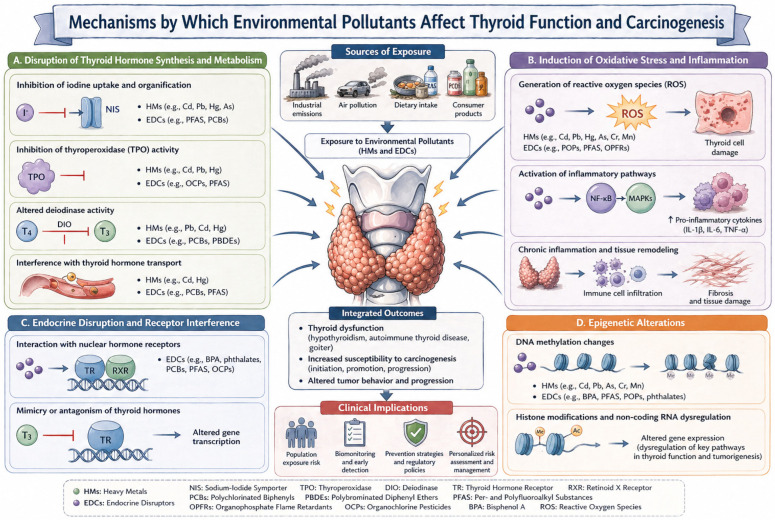
Schematic overview of the most important molecular mechanisms through which environmental pollutants may contribute to thyroid dysfunction and thyroid carcinogenesis. Environmental exposure to heavy metals (HMs) and endocrine-disrupting chemicals (EDCs) occurs through multiple sources. Once absorbed, these contaminants may interfere with thyroid hormone synthesis, metabolism, transport, and receptor signaling, leading to disruption of thyroid homeostasis. Major molecular mechanisms include inhibition of iodine uptake and thyroperoxidase activity, altered deiodinase function, oxidative stress, chronic inflammation, activation of intracellular signaling pathways, and epigenetic alterations such as DNA methylation changes and histone modifications.

Therefore, the biological effects of environmental pollutants on the thyroid appear to result from multiple converging mechanisms involving endocrine disruption, oxidative stress, inflammation, and epigenetic dysregulation, ultimately contributing to thyroid dysfunction and carcinogenesis, as reported in Figure 3.

## 5. Molecular Mechanisms Underlying Effects on Thyroid Dysfunction and Carcinogenesis

Numerous epidemiological studies suggest an association between exposure to environmental contaminants, thyroid dysfunction, and carcinogenesis-related processes; however, demonstrating a causal link and defining the underlying biological mechanisms remain challenging. Indeed, although these aspects have been investigated in various experimental models, the currently available evidence is still limited, especially in humans. This reflects the complexity of assessing exposure to a broad range of heterogeneous environmental contaminants as well as the difficulty of experimentally reproducing realistic scenarios, often characterized by chronic, low-dose, and mixed exposures. In light of these methodological limitations, experimental research has provided crucial insights into the multiple and interconnected molecular mechanisms underlying these processes. The most relevant findings currently available from in vitro and in vivo models are summarized in Table 8, Table 9, Table 10, Table 11 and Table 12, according to contaminant type (metals and EDCs) and exposure scenario (single compounds vs. mixtures). Although highly interconnected, these mechanisms can be schematically classified into key functional pathways, as detailed below.

### 5.1. Effects on Thyroid Hormone Biosynthesis and Activity

Among the most recurrent alterations, interference with thyroid hormone biosynthesis [247,258,264] and with the maintenance of the differentiated follicular phenotype emerges as a key event. In several experimental models, a variety of HMs (including Cd, As, and Hg) and EDCs have been shown to impair NIS-mediated iodine uptake and to alter the expression of essential genes for thyroid function and identity, including *Slc5a5/NIS*, *TPO*, *Tg*, *Pax8*, *Foxe1*, and *Nkx2-1* [251,252,258,261,262,263].

These alterations compromise both the biosynthetic capacity and the differentiated phenotype of thyrocytes, often leading to reduced hormone production and altered responsiveness to TSH. Structural and ultrastructural damage, including follicular disorganization and epithelial dedifferentiation, is frequently observed in parallel, supporting the link between endocrine disruption and tissue remodeling [251,280]. Consistently, experimental studies have shown that As exposure may induce direct cytotoxic effects in thyroid follicular cells, leading to reduced cell viability, morphological alterations, and increased apoptosis. These effects appear to be mediated, at least in part, by an imbalance in pro- and anti-apoptotic pathways, with an increased Bax/Bcl-2 ratio [248]. Furthermore, several EDCs and HMs have been associated with altered regulation of TSH secretion, likely reflecting disruption of neuroendocrine feedback mechanisms rather than direct effects on hypothalamic TRH synthesis [268,272,276].

Epidemiological and experimental evidence indicate that compounds such as PFAS, phthalates, and organochlorine pesticides may alter endocrine signaling and thyroid hormone regulation and interfere with thyroid hormone transport and metabolism, competing with T4 for binding to carrier proteins [300]. Similarly, phthalates and PFOS may affect peripheral thyroid hormone metabolism, potentially through modulation of deiodinase activity [207]. Finally, several contaminants, including PCBs and BPA, may interfere with thyroid hormone receptor (TR)-mediated signaling, leading to altered transcriptional responses even in the presence of normal circulating hormone concentrations [208].

### 5.2. Oxidative Stress and DNA Damage

Another major recurring mechanism is oxidative stress, which appears as a central hub linking thyroid dysfunction to carcinogenesis-related responses. In both in vitro and in vivo models, several contaminants increased ROS generation and altered antioxidant defenses, often in association with mitochondrial dysfunction and activation of redox-sensitive signaling pathways. For instance, BPA, PCB congeners, As, Cd, and Cr were all reported to induce oxidative imbalance in thyroid cells or tissues, accompanied by changes in NOX4, DUOX2, and lipid peroxidation [283]. In BPA-exposed FRTL-5 cells, ROS generation was also associated with indirect genotoxic effects, as suggested by impaired recovery from DNA damage and inhibition of genes involved in DNA replication and repair [254]. In other cases, as expected, oxidative stress was frequently associated at the same time with activation of MAPK, PI3K/AKT, JNK, or NFκB pathways, thereby contributing to cell proliferation, survival, inflammation, apoptosis, and, in some models, preneoplastic or tumor-promoting changes [247,248,249,250,251,252,253,254].

Furthermore, Cd and Pb compete with Se, an essential antioxidant trace element for thyroid homeostasis [29]. This competition thereby impairs the activity of Se-dependent enzymes (glutathione peroxidase) and the thyroid ability to neutralize reactive oxygen species, potentially promoting mutagenesis and carcinogenesis. Experimental studies suggest that Se may mitigate Cd-induced thyroid injury. In murine models, Se supplementation attenuated inflammatory responses and partially restored thyroid follicular architecture following Cd exposure. These findings highlight the role of oxidative stress in Cd-mediated thyroid toxicity and suggest the possibility that Se supplementation could have preventive relevance in populations chronically exposed to heavy metals [253].

### 5.3. Epigenetic Alterations

Increasing evidence indicates that both HMs and EDCs may induce persistent epigenetic changes, including alterations in DNA methylation, histone modifications, and non-coding RNA expression. Exposure to HMs and particulate matter has been associated with global and gene-specific DNA methylation changes, potentially affecting genes involved in thyroid differentiation and tumor suppression. These epigenetic modifications may represent a mechanistic link between chronic exposure and long-term disease risk, including the development of thyroid nodules and cancer [274].

### 5.4. Activation of Oncogenic Patterns

In TC models, BPA promoted proliferation through ER- and GPR30-related signaling and activation of AKT/mTOR pathway [250], while co-exposure to BPA and DEHP/MEHP was associated with HDAC6 upregulation, PTEN inhibition, AKT activation, and c-MYC upregulation [301]. Likewise, Cd and other metals promoted proliferation, migration, invasion, or cell-cycle progression through pathways involving GPER, ERK/AKT, and NFκB [247]. In stem/precursor thyroid cells, chronic exposure to low-dose tungsten or to metal mixtures stimulated proliferation, impaired differentiation, and induced preneoplastic features, further supporting the possibility that environmental pollutants may act not only as disruptors of thyroid homeostasis but also as facilitators of thyroid carcinogenesis-related processes [302]. In this context, stem/precursor thyroid cells, characterized by reduced antioxidant and detoxifying capacity compared to mature thyrocytes [303], may be more susceptible to environmental exposures, which in turn may more readily promote early carcinogenesis-related changes. Moreover, developmental stages also appear to represent particularly vulnerable windows of exposure. In vivo studies of prenatal, embryonic, and metamorphic exposure showed that environmental pollutants may alter thyroid morphology, hormone-related gene expression, and endocrine homeostasis, with consequences for growth, differentiation, skeletal ossification, neurodevelopment, and, in some cases, transgenerational outcomes [267,284,288,292].

Taken together, these findings indicate that disruption of thyroid hormone biosynthesis, structural and ultrastructural thyroid damage, oxidative stress, inflammation, epigenetic deregulation, altered hormone receptor signaling, and dysregulation of survival and proliferative pathways represent interconnected mechanisms through which environmental contaminants may contribute to both thyroid dysfunction and carcinogenesis. Such effects may be particularly relevant during vulnerable windows of exposure, such as the prenatal phase or the first years of life, since early interference with thyroid homeostasis may have long-term consequences on susceptibility to thyroid diseases.

## 6. Conclusions and Future Directions

The epidemiological evidence linking EDCs and HMs to thyroid cancer and thyroid dysfunctions remains heterogeneous and varies substantially across pollutant classes. The most consistent associations emerge for organochlorine pesticides and selected heavy metals, supported by both individual studies and pooled analyses, as well as by coherent biological mechanisms. On the contrary, the evidence for PFAS, bisphenols, and flame retardants is still limited or inconsistent, despite a solid mechanistic rationale suggesting potential thyroid-disrupting and carcinogenic effects. EDCs and HMs share several molecular pathways relevant to thyroid carcinogenesis. These include disruption of thyroid hormone synthesis and signaling, oxidative stress, DNA damage, and epigenetic alterations, which collectively may create a permissive environment for tumor initiation and progression, particularly under chronic, low-dose exposure conditions. However, the interpretation of these findings is constrained by important methodological limitations. Most available studies are observational and predominantly based on case–control designs. In addition, substantial heterogeneity in exposure assessment, population characteristics, and outcome definitions (especially concerning the definition of benign thyroid disease) contributes to uncertainty and limits causal inference. Another major limitation is the predominant focus on single compounds, whereas real-world exposure occurs as complex mixtures of pollutants with potentially additive or synergistic effects. Furthermore, although IARC classifications provide important information on general carcinogenic potential, they are not specific to TC and should be interpreted with caution in this context. Taken together, the available evidence supports a role for environmental pollutants in thyroid carcinogenesis, although the strength of this association differs across compounds and remains insufficient to establish definitive causal relationships for several classes. These findings highlight the need for well-designed prospective studies integrating improved exposure assessment, longitudinal follow-up, and mechanistic biomarkers, particularly during critical windows of susceptibility such as early life. From a translational perspective, the thyroid gland may represent a sensitive indicator of environmental exposure, given its intrinsic susceptibility to both endocrine disruption and oxidative damage. In this context, the concept of “molecular mimicry”—whereby HMs compete with essential elements such as iodine or Se—may offer a useful framework to better understand pollutant-thyroid interactions. This raises the possibility that targeted strategies aimed at optimizing micronutrient status or modulating metal bioavailability could mitigate, at least in part, the biological impact of environmental exposure. In highly contaminated areas, this concept may even support the exploration of preventive or therapeutic approaches aimed at reducing metal accumulation within the thyroid, although this remains a speculative and largely unexplored field requiring dedicated investigation. Future research integrating epidemiology, experimental biology, and emerging approaches such as exposomics and multi-omics will be essential to clarify these complex interactions and to move toward a more personalized and environmentally informed model of endocrine care.

## Figures and Tables

**Table 1:** Toxic mechanisms of Mercury (Hg), lead (Pb), Chromium (Cr), Cadmium (Cd), and Arsenic (As) [15].

Metal	Organ Toxicity	Disrupted Macromolecule/Mechanism of Action	Main Specific Thyroid Effects
Hg [45,46]	- CNS injuries	- Thiol binding (GSH conjugation)	Autoimmunity; thyroid hormone alterations; possible thyroid carcinogenesis [47,48]
	- Renal dysfunction	- Enzymes inhibition	
	- GI ulceration	- ROS production	
	- Hepatotoxicity	- Aquaporins mRNA reduction	
		- Glutathione peroxidase inhibition	
		- Increased c-fos expression	
Pb [49,50,51,52]	- CNS injury	- Increased inflammatory cytokines IL-1β, TNF-α, and IL-6 in the CNS	Disruption of the hypothalamus-pituitary-thyroid axis, TSH and thyroid hormone alterations; possible association with thyroid autoimmunity [53]
	- Lungs dysfunction	- Increased serum ET-1, NO, and EPO	
	-Hematological changes (Anemia)	- Inactivation of δ-ALAD and ferrochelatase (inhibition of heme biosynthesis)	
	- GI Colic	- Reduced GSH, SOD, CAT, and GPx levels	
	- Liver damage		
	- Reduced pulmonary function		
	- Cardiovascular dysfunction		
Cr [54,55]	- Kidney dysfunction	- DNA damage	Limited evidence regarding thyroid dysfunction; possible thyroid carcinogenic [56]
	- GI disorders	- Genomic instability	
	- Dermal diseases	- Oxidative stress and ROS generation	
	- Increasing the incidence of cancers including lungs, larynx, bladder, kidneys, testicular, bone, and thyroid		
Cd [57,58,59,60,61]	- Degenerative bone disease	- miRNA expression dysregulation	Impaired iodide uptake, TPO inhibition, altered deiodinase activity, and thyroid hormone metabolism; possible association with thyroid cancer [62,63]
	- Kidney dysfunction	- Apoptosis	
	- Liver damage	- Endoplasmic reticulum stress	
	- GI disorders	- Cd-MT absorption by the kidneys	
	- Lungs injuries	- Dysregulation of Ca, Zn, and Fe homeostasis	
	- Disorders in the metabolism of Zn and Cu	- Low serum PTH	
	- Cancer	- ROS generation	
		- Altered phosphorylation cascades	
As [64,65,66]	- Cardiovascular dysfunction	- Damage of capillary endothelium	Reduced thyroid hormone levels, increased TSH, and possible goiter; possible thyroid carcinogenesis [56]
	- Skin and hair changes	- Thiol binding (GSH conjugation)	
	- CNS injury	- Uncoupler of oxidative phosphorylation (inhibition of ATP formation)	
	- GI discomfort	- Alterations in neurotransmitter homeostasis	
	- Liver damage		

CNS: Central nervous system; GI: Gastrointestinal; GSH: Reduced glutathione; ROS: Reactive oxygen species.; mRNA: Messenger RNA; IL: Interleukin; TNF-α: Tumor necrosis factor alpha; ET-1: Endothelin-1; NO: Nitric oxide; EPO: Erythropoietin; δ-ALAD: Delta-aminolevulinic acid dehydratase; SOD: Superoxide dismutase; CAT: Catalase; GPx: Glutathione peroxidase; ATP: Adenosine triphosphate; miRNA: MicroRNA.

**Table 2:** Classification and Categorization of Persistent Organic Pollutants (POPs) under the Stockholm Convention. This table illustrates the categorization and chronological inclusion of 36 POPs under the Stockholm Convention, spanning from the original ‘Dirty Dozen’ in 2001 to the latest 2023–2025 amendments (COP-11 and COP-12) targeting emerging industrial chemicals and pesticides; substances are organized by primary source with their respective Convention reference numbers.

Category	No.	Chemical Substance	Listing Year
Pesticides	1–9	Original Pesticides (Aldrin, Chlordane, DDT, Dieldrin, Endrin, Heptachlor, Mirex, Toxaphene, HCB)	2001
Pesticides	13	Lindane	2009
Pesticides	14	Chlordecone	2009
Pesticides	22	Technical Endosulfan and isomers	2011
Pesticides	30	Dicofol	2019
Pesticides	31	Methoxychlor	2023
Pesticides	32	Chlorpyrifos	2025
Industrial Chemicals	10	PCB (Polychlorinated Biphenyls)	2001
Industrial Chemicals	18	PFOS, its salts, and PFOSF	2009
Industrial Chemicals	19	Hexabromobiphenyl (PBB)	2009
Industrial Chemicals	20–21	PBDEs (Tetra, Penta, Hexa, Hepta-BDE)	2009
Industrial Chemicals	23	HBCD (Hexabromocyclododecane)	2013
Industrial Chemicals	24	HCBD (Hexachlorobutadiene)	2015
Industrial Chemicals	25	PCNs (Polychlorinated Naphthalenes)	2015
Industrial Chemicals	26	SCCPs (Short-chain chlorinated paraffins)	2017
Industrial Chemicals	27	DecaBDE (Decabromodiphenyl ether)	2017
Industrial Chemicals	28	PFOA, its salts, and related compounds	2019
Industrial Chemicals	29	PFHxS, its salts, and related compounds	2022
Industrial Chemicals	33	Dechlorane Plus	2023
Industrial Chemicals	34	UV-328	2023
Industrial Chemicals	35	MCCPs (Medium-chain chlorinated paraffins)	2025
Industrial Chemicals	36	LC-PFCAs (Long-chain perfluorocarboxylic acids)	2025
By-products	11–12	PCDD (Dioxins) and PCDF (Furans)	2001
Mixed	15	Pentachlorobenzene	2009
Mixed	16–17	Alpha-HCH and Beta-HCH	2009

Abbreviations: POPs: Persistent Organic Pollutants; DDT: Dichlorodiphenyltrichloroethane; HCB: Hexachlorobenzene; HCH: Hexachlorocyclohexane; PCB: Polychlorinated Biphenyls; PBB: polybrominated biphenyls; PCDD: Polychlorinated dibenzo-p-dioxins; PCDF: Polychlorinated dibenzofurans; PFOS: perfluorooctane sulfonate; PFOSF: Perfluorooctane sulfonyl fluoride; PFOA: Perfluorooctanoic acid; PFHxS: Perfluorohexane sulfonic acid; PBDE: Polybrominated diphenyl ethers; SCCPs: Short-chain chlorinated paraffins; MCCPs: Medium-chain chlorinated paraffins; LC-PFCAs: Long-chain perfluorocarboxylic acids.

**Table 3:** Environmental occurrence and regulatory status of major endocrine-disrupting chemicals and pollutants potentially associated with TC. Corresponding epidemiological studies investigating thyroid cancer risk and related references are reported in Table 4.

Pollutant Class	Compound	Major Sources/ Environmental Occurrence	Regulatory Status	* Environmental Persistence	Main Human Exposure Routes
PFAS	PFOA	Industrial emissions, contaminated drinking water, food packaging, non-stick cookware, water- and oil-repellent materials	Restricted/banned in several countries	Very persistent	Drinking water, food, and dermal contact
PFAS	PFOS	Firefighting foams, industrial products	Restricted under international conventions	Very persistent	Water, food, dust, and dermal contact
Organochlorine pesticides	DDT/DDE	Agricultural pesticides (historical use)	Banned in many countries	Very persistent	Food chain
Persistent organic pollutants (POPs)	PCBs	Industrial electrical equipment, contaminated soil, and sediments	Banned in many countries	Highly persistent	Food, occupational exposure
Flame retardants	PBDEs	Furniture, electronics, household dust, hydraulic fluids, and flame retardants.	Restricted in several countries	Persistent	Indoor dust, animal-derived food
Plasticizers	Phthalates (DEHP, DBP)	Plastics, cosmetics, consumer products, food packaging	Restricted in some products	Moderate persistence	Food, dermal exposure
Bisphenols	BPA	Plastic containers, food packaging, epoxy resins, food-contact materials, thermal paper	Restricted in some food-contact materials	Moderate persistence	Food, beverages, dermal exposure, inhalation
Heavy metals	Manganese	Mining; steel and alloy production; battery manufacturing; fuel additives; agricultural fungicides	Regulated in drinking water, occupational exposure limits established	Moderate persistence	Food, drinking water; occupational exposure; minor dermal exposure
Heavy metals	Mercury	Found in air, water, and soil (natural sources: volcanoes, geothermal activity)	Strictly regulated; limits in food (fish), drinking water, and occupational settings	Highly persistent	Food, occupational exposure; minor dermal exposure
Heavy metals	Cadmium	Industrial emissions, cigarette smoke	Still present in environment	Persistent	Food, smoking
Heavy metals	Arsenic	Contaminated drinking water	Regulated but still present in some areas	Persistent	Drinking water
Environmental contaminants	Nitrate	Fertilizers, agricultural runoff	Not banned (regulated levels)	Variable	Drinking water

* Very Persistent: Years to decades; Highly Persistent: Months to years; Persistent: Months; Moderately Persistent: Days to weeks/months. Abbreviations: PFAS: per- and polyfluoroalkyl substances; PFOA: perfluorooctanoic acid; PFOS: perfluorooctane sulfonate; DDT: dichlorodiphenyltrichloroethane; DDE: dichlorodiphenyldichloroethylene; POPs: persistent organic pollutants; PCBs: polychlorinated biphenyls; PBDEs: polybrominated diphenyl ethers; DEHP: di(2-ethylhexyl) phthalate; DBP: dibutyl phthalate; BPA: bisphenol A.

**Table 4:** Epidemiological evidence and proposed mechanisms linking environmental pollutants to thyroid cancer. The table summarizes the current epidemiological evidence linking environmental pollutants to thyroid cancer. For each pollutant class and compound, the table reports the level of epidemiological evidence with data from different types of epidemiological studies, including meta-analyses, cohort nested case–control studies, and conventional case–control studies. The principal biological mechanisms proposed to explain thyroid carcinogenesis, shared across multiple pollutant classes, include endocrine disruption, oxidative stress, DNA damage, epigenetic alterations, and interference with thyroid hormone synthesis, transport, and receptor signaling. The inclusion of the International Agency for Research on Cancer (IARC) classification provides an additional layer of interpretation by indicating the overall carcinogenic hazard of each compound. However, it should be emphasized that IARC classifications refer to general carcinogenicity and are not specific to thyroid cancer. Therefore, they should be interpreted as an indicator of broader oncogenic potential rather than direct evidence of thyroid carcinogenicity. * Di(2-ethylhexyl) phthalate. Group: 1 = Carcinogenic to humans; 2A = probably carcinogenic to humans; 2B = possibly carcinogenic to humans; 3 = Not classifiable as to its carcinogenicity to humans; IARC MONOGRAPHS ON THE IDENTIFICATION OF CARCINOGENIC HAZARDS TO HUMANS https://monographs.iarc.who.int/agents-classified-by-the-iarc/ accessed on 25 April 2026.

Pollutant Class	Compound	Epidemiological Evidence for TC	Key Studies	Proposed Thyroid Carcinogenic Mechanisms	IARC Classification Group
PFAS	PFOA	Limited/inconsistent evidence	Barry 2013 [199] van Gerwen 2024 [200]	Thyroid hormone transport disruption, endocrine signaling alteration	1
PFAS	PFOS	Limited evidence	Yang 2025 [201] Wang 2025 [202]	Thyroid hormone metabolism interference	2B
Organochlorine pesticides	DDT/DDE	Positive associations in several studies	Aschebrook-Kilfoy 2011 [203] Han 2019 [204] Rusiecki 2024 [205]	Endocrine disruption, oxidative stress	2A
Persistent organic pollutants	PCBs	Mixed evidence	Aschebrook-Kilfoy 2011 [203] Yang 2023 [63]	Interaction with thyroid hormone receptors	1 (some congeners)
Flame retardants	PBDEs	Positive associations	Zhang 2021 [206] Yang 2023 [63]	Disruption of thyroid hormone transport proteins	2A
Plasticizers	* Phthalates	Moderate evidence	Liu 2020 [207] Yang 2023 [63]	Alteration of thyroid hormone metabolism	2B
Bisphenols	BPA	Limited evidence	Zhang 2023 [208] Chen 2022 [209]	Thyroid receptor interference	3
Heavy metals	Manganese	Positive associations reported	van Gerwen 2022 [210]	May interfere with cellular antioxidant defense systems	Not listed as carcinogen
Heavy metals	Mercury	Positive association	Webster 2024 [211]	RAS, MAPK, Akt activation, cell proliferation, DNA damage	2B/3
Heavy metals	Cadmium	Moderate evidence	Park 2020 [212]	Oxidative stress, DNA damage	1
Heavy metals	Arsenic	Limited-moderate evidence	He 2022 [213] van Gerwen 2022 [210]	Epigenetic alterations, oxidative stress	1
Environmental contaminants	Nitrate	Moderate evidence	Ward 2010 [214] Bahadoran 2015 [215]	Inhibition of iodide uptake (NIS)	2A

Abbreviations: PFAS: per- and polyfluoroalkyl substances; PFOA: perfluorooctanoic acid; PFOS: perfluorooctane sulfonate; TC: thyroid cancer; DDT: dichlorodiphenyltrichloroethane; DDE: dichlorodiphenyldichloroethylene; PCBs: polychlorinated biphenyls; PBDEs: polybrominated diphenyl ethers; BPA: bisphenol A; MAPK: mitogen-activated protein kinase; Akt: protein kinase B; NIS: sodium/iodide symporter; IARC: International Agency for Research on Cancer.

**Table 5:** Representative studies assessing the association between heavy metal (HM) exposure and thyroid cancer (TC) phenotype, including cross-sectional, cohort, and meta-analytic designs across different populations.

Year	Authors (Ref.)	Study Design	Population	Sample Size	Metals
2015	Chung [227]	Cross-sectional	Korea	92	Cd, Pb, Se, Zn, Hg
2019	Deng [55]	meta-analysis	14 Countries	19109	Cr (VI)
2021	Hu [229]	Cross-sectional	China	608	Co, Cr, Cu, Fe, Mn, Mo, Se, Sr, Zn, I
2025	Capezzone [230]	Cohort	Italy	3210	Not mentioned

Abbreviations: Cd: Cadmium; Pb: Lead; Se: Selenium; Zn: Zinc; Hg: Mercury; Cr: Chromium; Co: Cobalt; Cu: Copper; Fe: Iron; Mn: Manganese; Mo: Molybdenum; Sr: Strontium; I: Iodine.

**Table 6:** Key meta-analyses on environmental pollutants and thyroid cancer or thyroid-related outcomes (updated through 2025).

Authors	Year	Pollutant	Compounds Evaluated	Outcome	Main Findings
Han et al. [204]	2019	Pesticides/POPs	Pesticides, herbicides, POPs	Thyroid cancer risk	Positive association reported for pesticide exposure, with stronger signals for herbicides/agricultural exposure.
van Gerwen et al. [210]	2022	Heavy metals	Multiple metals, including Mn, Co, Cd, Pb, Hg	Thyroid cancer risk	Heterogeneous evidence; some metal-specific associations reported, but no uniform pattern across all metals.
Yang et al. [63]	2023	Mixed EDCs	PCBs, PBDEs, phthalates, BPA, heavy metals	Thyroid cancer risk	Positive associations reported for PBDEs, phthalates, and heavy metals; BPA not significant; PCB findings mixed.
van Gerwen et al. [200]	2024	PFAS	PFOA, PFOS, PFNA, PFHxS	Thyroid cancer risk	Evidence remained limited and inconsistent.
Sassano et al. [235]	2025	PFAS	Multiple PFAS	Thyroid cancer risk	No positive associations for measured serum levels of six PFAS overall; a positive pooled signal was reported for PFOS in a nested case–control analysis.
Wang et al. [202]	2025	PFAS	PFOA, PFOS, PFNA, and other PFAS	Thyroid cancer risk	Overall association between PFAS exposure and thyroid cancer was not statistically significant.
Yang et al. [201]	2025	Pesticides	Insecticides, herbicides, fungicides	Thyroid cancer risk	Positive associations reported across pesticide subclasses, with stronger female-specific associations.

Only meta-analyses or systematic reviews with pooled quantitative estimates directly relevant to thyroid cancer or thyroid-related outcomes are included. Abbreviations: POPs: Persistent Organic Pollutants; EDCs: Endocrine-Disrupting Chemicals; PCBs: Polychlorinated Biphenyls; PBDEs: Polybrominated Diphenyl Ethers; BPA: Bisphenol A; PFAS: Per- and Polyfluoroalkyl Substances; PFOA: Perfluorooctanoic Acid; PFOS: Perfluorooctane Sulfonate; PFNA: Perfluorononanoic Acid; PFHxS: Perfluorohexane Sulfonic Acid; Mn: Manganese; Co: Cobalt; Cd: Cadmium; Pb: Lead; Hg: Mercury.

**Table 7:** Early-life exposure to environmental pollutants and potential risk of thyroid cancer.

Authors	Year	Exposure Window	Pollutant/Factor	Study Type	Main Findings	Relevance for Thyroid Carcinogenesis
Kitahara et al. [239]	2021	In utero/perinatal	Maternal health conditions (e.g., diabetes, thyroid disease)	Nested case–control (Nordic registries)	Associations observed with high birth weight, congenital hypothyroidism, and maternal metabolic/thyroid conditions	Strong evidence supporting early-life susceptibility
Thi-Van-Trinh Tran et al. (Sister Study) [240]	2025	In utero/early life	Gestational diabetes, hypertensive disorders, birth weight	Prospective cohort	Positive associations with gestational diabetes, hypertensive disorders, and higher birth weight	Supports role of prenatal metabolic environment
Deziel et al. [241]	2021	Perinatal/childhood	Birth characteristics	Population-based case–control (pediatric thyroid cancer)	Birth factors associated with pediatric thyroid cancer risk	Suggests early-life determinants of thyroid carcinogenesis
Coperchini et al. [242]	2021	Prenatal/childhood	PFAS	Review (mechanistic + epidemiological)	PFAS alter thyroid hormone homeostasis during development	Strong biological plausibility, limited cancer-specific evidence
Ballesteros et al. [243]	2017	Prenatal/childhood	PFAS	Systematic review	Associations with altered thyroid hormone levels	Supports endocrine-disrupting effects in early life
Leemans et al. [244]	2019	Prenatal/early life	Pesticides	Review	Many pesticides disrupt thyroid hormone signaling	Mechanistic support for carcinogenic pathways
Alsen et al. [233]	2021	General (including early-life)	EDCs (PCBs, PBDEs, BPA, phthalates)	Review	Associations between EDC exposure and thyroid cancer suggested	Evidence indirect; limited early-life-specific data
Norouzi et al. [245]	2023	Mainly adult (limited early-life data)	Pesticides	Systematic review	Positive associations between pesticide exposure and thyroid cancer	Limited direct early-life evidence
Iglesias et al. [237]	2017	Childhood	Ionizing radiation	Review	Strong association with thyroid cancer, especially in children	Strongest evidence for early-life environmental exposure
Nikiforov [238]	2006	Childhood	Ionizing radiation	Review	Radiation induces genetic alterations (e.g., RET/PTC)	Established causal mechanism in thyroid carcinogenesis

Abbreviations: PFAS: Per- and Polyfluoroalkyl Substances; EDCs: Endocrine-Disrupting Chemicals; PCBs: Polychlorinated Biphenyls; PBDEs: Polybrominated Diphenyl Ethers; BPA: Bisphenol A.

**Table 8:** In vitro evidence of heavy metal (HM)-induced alterations related to thyroid dysfunction and carcinogenesis.

Metals	Cell Model	Dose	Exposure Time	Main Effects and Underlying Mechanisms	Ref.
Arsenic	Rat thyroid follicular cell line	NaAsO_2_: 0.4, 0.8, and 3.2 μM	24 h	Reduced T3 and T4 levels and altered ERα and TRα mRNA expression, accompanied by decreased Keap1 and AKT mRNA expression and increased Nrf2 and PI3K mRNA expression	[247]
	Nthy ori 3–1 normal human thyroid follicular epithelial cell line	NaAsO_2_: 0.1, 1, 10, 50, 100 μM	12, 24, and 48 h	Reduced cell viability, induced morphological changes, increased LDH release, and promoted apoptosis in a dose- and time-dependent manner, accompanied by decreased Bcl-2 expression and increased Bax expression	[248]
Cadmium	Nthy ori 3–1 normal human thyroid follicular epithelial cell line	CdCl_2_: 0.1 to 100 μM	24, 48, and 72 h	Reduced cell growth in a dose- and time-dependent manner, accompanied by decreased phospho-ERK levels, increased ROS production and lipid peroxidation, and induction of ER stress, as indicated by increased GRP78 expression; these effects were attenuated by quercetin	[249]
	WRO and FRO human thyroid cancer cells	CdCl_2_: 250, 500, 750, and 1000 nM	12, 48, and 72 h	Promoted cell proliferation, invasion, and migration through activation of the GPER/ERK/AKT/NF-κB signaling pathway, with increased cyclin A and cyclin D1 expression and IL-8 secretion	[250]
Tungsten	Human primary mature and immature thyrocytes, and thyrocytes differentiated from tungsten-exposed immature thyrocytes	Na_2_WO_4_: 30 nM	15 days	Promoted growth, reduced apoptosis, impaired differentiation, and induced preneoplastic transformation in thyroid stem/precursor cells, accompanied by abnormal expression of thyroid cancer-related genes, activation of H2AX and 53BP1, and ERK pathway activation	[251]

Abbreviations: AKT: Protein kinase B; Bax: Bcl-2-associated X protein; Bcl-2: B-cell lymphoma 2; CdCl_2_: Cadmium chloride; ER: Endoplasmic reticulum; ERα: Estrogen receptor alpha; ERK: Extracellular signal-regulated kinase; GPER: G protein-coupled estrogen receptor; GRP78: Glucose-regulated protein 78; H2AX: H2A histone family member X; IL-8: Interleukin-8; Keap1: Kelch-like ECH-associated protein 1; LDH: Lactate dehydrogenase; Na_2_WO_4_: Sodium tungstate; NaAsO_2_: Sodium arsenite; NF-κB: Nuclear factor kappa B; Nrf2: Nuclear factor erythroid 2–related factor 2; PI3K: Phosphoinositide 3-kinase; ROS: Reactive oxygen species; T3: Triiodothyronine; T4: Thyroxine; TRα: Thyroid hormone receptor alpha.

**Table 9:** In vitro evidence of endocrine-disrupting chemical (EDC)-induced alterations related to thyroid dysfunction and carcinogenesis.

EDCs	Cell Model	Dose	Exposure Time	Main Effects and Underlying Mechanisms	Ref.
BPA	PCCL3 rat thyroid follicular cell line	10^−9^, 10^−7^, 10^−5^, and 10^−3^ M	24 h	Increased intracellular H_2_O_2_ production, decreased Nis mRNA expression levels, and increased Duox2 mRNA expression levels, associated with oxidative stress	[252]
	Nthy ori 3–1 normal human thyroid follicular epithelial cell line	0.1 and 0.5 μM	24 and 48 h	Enhanced cell proliferation, increased ROS generation and NOX4 expression, and activation of the MAPK and PI3K/AKT pathways	[253]
	FRTL-5 rat thyroid follicular cell line	10^−9^ M	1, 3, and 7 days	Increased H_2_O_2_ generation, impaired DNA damage recovery, and inhibition of genes involved in DNA replication and repair, suggesting indirect genotoxic activity	[254]
	GH3 rat pituitary epithelial tumor cell line	10^−9^ to 10^−5^ M	48 h	Decreased T3-induced prolactin production through non-competitive interaction with thyroid hormone receptors	[255]
	MDA-T32, BCPAP, and TPC-1 human thyroid cancer cell lines in 3D culture	10^−9^, 10^−6^, 10^−5^, and 10^−4^ M	24 and 48 h	Induced an EMT-like phenotype, characterized by decreased E-cadherin and increased vimentin expression, along with reduced TG secretion	[256]
	TPC-1 and BCPAP human thyroid cancer cell lines	0.1 and 0.5 μM	24 and 48 h	Enhanced cell proliferation, migration, and invasion, accompanied by increased ROS generation and NOX4 expression, activation of the MAPK and PI3K/AKT pathways, and increased growth of BCPAP xenografts	[253]
	Nthy-BRAFV600E cell line	10^−10^ to 10^−3^ M	24, 48, and 72 h	Enhanced BRAFV600E-induced cell proliferation, migration, invasion, and EMT through the ERK-COX2 signaling pathway	[257]
	FRTL-5 rat thyroid follicular cell line	0.03 to 300 μM	24 and 48 h	Inhibited iodide uptake in a concentration-dependent manner through non-competitive inhibition of Nis, and altered the expression of thyroid-specific genes involved in TH synthesis and transcriptional regulation, including *Slc5a5*, *Tpo*, *Tg*, *Pax8*, *Foxe1*, and *Nkx2-1*, without affecting TPO activity	[258]
	BHP10-3 human thyroid cancer cell line	BPA: 1 mM to 10 nM; 17β-estradiol (E2): 0.1 mM to 1 nM	24, 48, and 72 h	Enhanced cell proliferation, migration, and invasion, accompanied by increased Erα, Erβ, and GPR30 expression and rapid activation of the AKT/mTOR pathway	[259]
Triclosan	FRTL-5 rat thyroid follicular cell line	0.03 to 300 μM	24 and 48 h	Inhibited Nis-mediated iodide uptake in a concentration-dependent manner through non-competitive inhibition of Nis, and inhibited TPO activity, without significantly altering the expression of thyroid-specific genes	[258]
Triclocarban	FRTL-5 rat thyroid follicular cell line	0.03 to 300 μM	24 and 48 h	Inhibited Nis-mediated iodide uptake in a concentration-dependent manner through non-competitive inhibition of Nis, and inhibited TPO activity, without significantly altering the expression of thyroid-specific genes	[258]
BDE-47	FRTL-5 rat thyroid follicular cell line	0.03 to 300 μM	24 and 48 h	Inhibited Nis-mediated iodide uptake in a concentration-dependent manner through non-competitive inhibition of Nis, decreased Tpo expression, and did not affect TPO activity	[258]
	Nthy ori 3–1 normal human thyroid follicular epithelial cell line	25, 50, 100, 200, 400, and 800 μM	24 h	Reduced cell viability, impaired cell-cycle progression, enhanced migration, and decreased invasion, associated with binding to BRAF and activation of the MEK-ERK signaling pathway, along with reduced NIS mRNA expression	[260]
PCB118	Primary culture of normal human thyroid cells	0.025, 0.25, 2.5, 25, 250, 2500, and 25,000 nM	24, 48, or 72 h	Decreased TG and T4 levels and reduced NIS expression, accompanied by increased Akt and p-FoxO3a signaling, consistent with activation of the Akt/FoxO3a/NIS pathway	[261]
	FRTL-5 rat thyroid follicular cell line	0.25, 2.5, and 25 nM	24 h	Altered expression of genes involved in the mitochondrial respiratory chain, increased ROS generation, and reduced Nis expression, associated with mitochondrial damage and activation of the JNK pathway	[262]
PCB118 and PCB126	Primary culture of normal human thyroid cells	2.5 and 5 μM	24 h	Increased IL-1β and IL-6 mRNA and protein expression, reduced TG and NIS mRNA expression, and increased ROS generation, accompanied by activation of the AhR and Nrf2 pathways	[263]
Aroclor 1254	GH3 rat pituitary epithelial tumor cell line	10^−11^ to 10^−6^ M	48 h	Enhanced T3-mediated prolactin production through competitive binding to thyroid hormone receptors	[255]
TCDD	GH3 rat pituitary epithelial tumor cell line	10^−12^ to 10^−6^ M	48 h	Inhibited T3-TR binding in a dose-dependent manner through competitive interaction with thyroid hormone receptors	[255]
Benzo[a]pyrene	Human embryonic stem cell-derived thyroid organoids	10 μM	Sex hormone mixtures for 3 days, followed by 24 h exposure	Downregulated TG expression and induced transcriptomic changes related to oxidative phosphorylation, ROS response, apoptosis, and cell-cycle regulation, together with upregulation of CYP1A1, CYP1B1, and NQO1; in the male hormonal context, it also promoted lipid metabolism-, inflammation-, and NF-κB-related gene expression	[264]
PCB153	Human embryonic stem cell-derived thyroid organoids	10 μM	Sex hormone mixtures for 3 days, followed by 24 h exposure	Exerted modest transcriptomic effects in the presence of the male hormone mix, whereas no significant changes were observed under the female hormone condition	[264]

Abbreviations: AhR: Aryl hydrocarbon receptor; AKT: Protein kinase B; BPA: Bisphenol A; COX2: Cyclooxygenase-2; Duox2: Dual oxidase 2; E2: 17β-estradiol; EMT: Epithelial–mesenchymal transition; ERK: Extracellular signal-regulated kinase; FoxO3a: Forkhead box O3a; GPR30: G protein-coupled estrogen receptor 30; H_2_O_2_: Hydrogen peroxide; IL: Interleukin; JNK: c-Jun N-terminal kinase; MAPK: Mitogen-activated protein kinase; MEK: Mitogen-activated protein kinase kinase; mTOR: Mammalian target of rapamycin; NF-κB: Nuclear factor kappa B; NIS/Nis: Sodium-iodide symporter; NOX4: NADPH oxidase 4; NQO1: NAD(P)H quinone dehydrogenase 1; Nrf2: Nuclear factor erythroid 2–related factor 2; PCB: Polychlorinated biphenyl; PI3K: Phosphoinositide 3-kinase; ROS: Reactive oxygen species; T3: Triiodothyronine; T4: Thyroxine; TCDD: 2,3,7,8-Tetrachlorodibenzo-p-dioxin; TG/Tg: Thyroglobulin; TH: Thyroid hormone; TPO/Tpo: Thyroperoxidase; TR: Thyroid hormone receptor.

**Table 10:** In vitro and in vivo evidence of thyroid dysfunction- and carcinogenesis-related alterations induced by mixtures or combinations of heavy metals (HMs) and endocrine disrupting chemicals (EDCs).

EDCs/Metals Mixtures or Combinations	Animal Model	Dose	Exposure Time	Main Effects and Underlying Mechanisms
PCBs (Aroclor 1254) and Pb [265]	Male albino Wistar rats	PCBs: 0.25, 0.5, and 1 mg/kg/d; Pb: 0.1, 0.5, and 1 mg/kg/d (3 × 3 dose design)	28 days	Altered thyroid homeostasis, with increased serum FT4, total and free T3, and TSH levels, decreased relative thyroid weight, increased total oxidative status and sulfhydryl groups, and decreased total antioxidant status, indicating disruption of oxidative-antioxidative balance along the hypothalamic-pituitary-thyroid axis
B, Cd, and Mo [266]	Female Wistar rats	Boric acid: 9.14 mg/L; cadmium chloride: 0.66 μg/L; ammonium heptamolybdate tetrahydrate: 1.286 mg/L, in drinking water	5 and 10 months	Accelerated the appearance of histological features of thyroid transformation, accompanied by decreased thyroid iodine content, in hypothyroid rats exposed to low environmental concentrations of B, Cd, and Mo
Cd and Pb [267]	*Bufo gargarizans* embryos/froglets		From embryo stage throughout metamorphosis	Induced thyroid histological alterations, reduced body size at metamorphosis, impaired skeletal ossification, and decreased jumping ability, accompanied by dysregulation of genes involved in thyroid hormone action and endochondral ossification; the Cd/Pb mixture exerted more severe effects than single-metal exposure.
Pb and F [268]	Zebrafish	Sodium fluoride (NaF): 80 mg/L; Pb (CH3COO)_2_: 60 mg/L	45 and 90 days	Induced thyroid histopathological alterations, oxidative stress, downregulation of T3 and T4, and time-dependent changes in thyroid endocrine-related gene expression; combined F and Pb exposure exerted mainly additive toxic effects and aggravated thyroid hormone reduction compared with single exposure
BDE-209 and Pb	Zebrafish [269]	BDE-209: 1, 10, and 100 μg/L; Pb: 10 μg/L	3 months	Parental co-exposure induced reproductive and thyroid endocrine disruption in adults and transgenerational developmental neurotoxicity in offspring, accompanied by increased transfer of PBDEs and Pb to eggs and enhanced debromination of BDE-209 to BDE-197
	Zebrafish [270]	BDE-209: 50, 100, and 200 μg/L; Pb: 5, 10, and 20 μg/L	From 2 to 144 h post-fertilization	Co-exposure elicited synergistic effects on whole-body T3 and T4 levels, accompanied by enhanced Pb uptake and reduced bioconcentration and metabolism of BDE-209, suggesting increased toxicological effects of the mixture
Pb and chlorpyrifos [271]	Wistar rats	Pb: 250 mg/kg; CPF: 4.25 mg/kg; Vitamin C: 100 mg/kg	9 weeks	Co-exposure induced thyroid dysfunction, with decreased serum T3 and T4 levels, increased TSH levels, and increased malondialdehyde levels, indicating oxidative stress; these effects were attenuated by vitamin C
TBT and BPS [272]	Wistar rats (8–10-week-old female)	TBT: 100 ng/kg/d; BPS: 50 μg/kg/d	15 days	Co-exposure deregulated the HPT axis, with increased T4, decreased T3, reduced TPO activity, increased TSHr mRNA expression, altered D1 and D2 expression in the HPT axis and liver, and thyroid histomorphological changes, accompanied by increased Nrf2/Keap1 expression, reduced thiol groups, and increased SOD activity
Cd and TBT [273]	Adult zebrafish and F1/F2 offspring	Cd: 100 ng/L; TBT: 100 ng/L	90 days in F0, with continued exposure in F1 and F2 generations	Co-exposure induced multigenerational thyroid endocrine disruption and developmental neurotoxicity, accompanied by decreased thyroid hormone levels, downregulation of hypothalamus-pituitary-thyroid axis-related genes, reduced dopamine and serotonin levels, and inhibited acetylcholinesterase activity
Cu, Hg, Pd, W, and Zn [219]	Human immature and differentiated thyroid cells	Nanomolar range	acute timing and 72 h	Increased proliferation of thyroid immature cells, with a greater effect for the metal mixture, and induced morphological alterations in immature cells, whereas no effects were observed in differentiated thyrocytes; these responses followed a biphasic hormetic pattern and were mediated by ERK1/2 activation
BPA and MEHP [274]	BCPAP human thyroid cancer cell line	10^−10^ to 5 × 10^−5^ M	24 and 48 h	Induced cell proliferation, accompanied by HDAC6 upregulation, H3K9ac-mediated PTEN inhibition, AKT activation, and c-MYC upregulation.
BPA + DEHP [274]	Sprague Dawley rats (3–4-week-old female)	DEHP: 150 mg/kg/d; BPA: 20 mg/kg/d; DEHP and BPA mixture	30 weeks	Co-exposure increased susceptibility to thyroid carcinogenesis, accompanied by HDAC6 upregulation, PTEN suppression, and c-MYC activation.
BPA + PS-NPs [275]	C57BL/6 mice (4-week-old male)	BPA: 50 mg/kg; PS-NPs: 5, 15, and 50 mg/kg	4 weeks	Disrupted thyroid hormone secretion and induced thyroid structural damage, with additive toxicity upon co-exposure to PS-NPs, which enhanced BPA-induced inhibition of ECM-related genes, including Col1a2, Col5a1, and Col5a2
BPA + DEHP [276]	Sprague Dawley rats (3-week-old)	DEHP: 150, 750 mg/kg/d; BPA: 20, 100 mg/kg/d	40 days	Altered serum TH and TSH levels, reduced thyroid weight, and increased the number of thyroid follicular epithelial cells; DEHP-induced CREB phosphorylation via estrogen receptor α (Esr1) was abolished by concomitant BPA exposure

Abbreviations: AKT: Protein kinase B; B: Boron; BDE-209: Decabromodiphenyl ether; BDE-197: Heptabromodiphenyl ether; BPA: Bisphenol A; BPS: Bisphenol S; Cd: Cadmium; CPF: Chlorpyrifos; CREB: cAMP response element-binding protein; Cu: Copper; D1: Deiodinase type 1; D2: Deiodinase type 2; DEHP: Di(2-ethylhexyl) phthalate; ECM: Extracellular matrix; ERK1/2: Extracellular signal-regulated kinase 1/2; FT4: Free thyroxine; H3K9ac: Histone H3 lysine 9 acetylation; HDAC6: Histone deacetylase 6; Hg: Mercury; HPT: Hypothalamic-pituitary-thyroid; MEHP: Mono(2-ethylhexyl) phthalate; Mo: Molybdenum; Pb: Lead; PCB: Polychlorinated biphenyl; PS-NPs: Polystyrene nanoplastics; PTEN: Phosphatase and tensin homolog; SOD: Superoxide dismutase; T3: Triiodothyronine; T4: Thyroxine; TBT: Tributyltin; TH: Thyroid hormone; TPO: Thyroperoxidase; TSH: Thyroid-stimulating hormone; TSHr: Thyroid-stimulating hormone receptor; W: Tungsten; Zn: Zinc.

**Table 11:** In vivo evidence of heavy metal-induced alterations related to thyroid dysfunction and carcinogenesis.

Metal	Animal Model	Dose	Exposure Time	Main Effects and Underlying Mechanisms	Ref.
Arsenic	Wistar rats (1-month-old male and female)	NaAsO_2_: 0.8, 4.0, and 20.0 mg/kg/d	20 weeks	Induced dose-dependent histopathological thyroid changes, increased serum E2 levels, and altered thyroid hormone metabolism, with decreased Tg, T3, and T4 levels, accompanied by altered ERα and TRα expression and dysregulation of the PI3K/Nrf2 pathway	[247]
	Sprague Dawley rats (3-week-old male and female)	NaAsO_2_: 10 mg/kg/d; TAK-242: 0.5 mg/kg/d; active vitamin D: 10 μg/kg/d	36 weeks; TAK-242 and active vitamin D from week 12	Induced thyroid dysfunction through activation of the TLR4/NF-κB pathway, leading to thyroid cell apoptosis, inflammatory damage, decreased serum TT3, FT3, TT4, and FT4 levels, and increased TSH and TgAb levels; these effects were mitigated by vitamin D and TLR4 inhibition	[277]
	Sprague Dawley rats	NaAsO_2_: 2.5, 5.0, and 10.0 mg/kg/d	12, 24, and 36 weeks	Induced arsenic accumulation in thyroid tissue and upregulated NLRP3 inflammasome-related proteins, leading to thyroid cell pyroptosis, inflammatory injury, epithelial-mesenchymal transition, fibrotic changes, and thyroid dysfunction, with decreased serum TT3, FT3, TT4, and FT4 levels and increased TSH levels	[278]
	Sprague Dawley rats	NaAsO_2_: 2.5, 5.0, and 10.0 mg/kg/d	36 weeks	Induced arsenic accumulation in thyroid tissue and histopathological thyroid damage, leading to decreased serum T3 and T4 levels and increased TSH levels, accompanied by an increased Bax/Bcl-2 ratio	[248]
	Adult guinea pigs	50 ppm arsenic as sodium arsenite or arsenic trioxide	11 weeks	Reduced serum thyroid hormone levels, increased tissue arsenic accumulation, decreased erythrocytic antioxidant defenses, and increased lipid peroxidation	[279]
	Zebrafish	1 and 10 mg/L of Sodium Arsenate	10, 30, 60, and 90 days	Induced transient thyroid histopathological alterations, including follicular cell hypertrophy, hyperplasia, and colloid depletion, although evidence of thyroid-disrupting activity remained equivocal	[280]
Cadmium	Pigs (6-week-old male)	CdCl_2_: 20 mg/kg	40 days	Induced thyroid inflammatory injury, fibrosis, endoplasmic reticulum stress, and apoptosis, accompanied by activation of antioxidant defenses (SOD1, SOD2, CAT, and GSH) and regulation of the miR-494-3p/PTEN axis	[281]
	Adult male Kunming mice	CdCl_2_: 30 mg/kg/d; fucoxanthin: 10, 25, and 50 mg/kg/d	CdCl_2_ for 30 days, followed by fucoxanthin treatment for 14 days	Decreased serum T3 and T4 levels and induced oxidative stress, structural thyroid abnormalities, and apoptosis, accompanied by activation of the ERK1/2 pathway and endoplasmic reticulum stress; these effects were attenuated by fucoxanthin	[282]
	Male C57 BL/6J mice	CdCl_2_: 2 mg/kg/d; MI: 360 mg/kg/d; Se: 0.2 or 0.4 mg/kg/d	14 days	Induced structural and inflammatory thyroid damage, with reduced follicular area, increased epithelial height and stroma, and increased MCP-1 and CXCL10 expression; these effects were attenuated by myo-inositol and seleno-L-methionine, especially in combination	[283]
	Pregnant rats and offspring	Cd: 1 and 2 mg/kg; Se: 1 and 2 mg/kg	Single subcutaneous administration on gestational day 6	Induced developmental thyroid alterations, characterized by microfollicle formation resembling thyrotoxicosis, following maternal cadmium exposure; these effects were attenuated by low-dose Se	[284]
Chromium	Wistar rats	Potassium dichromate (CrVI): 75 mg/L; Aronia melanocarpa extract: 2.5%; Hypericum perforatum extract: 2.5%	CrVI for 3 months, followed by 1 month of distilled water or plant extract treatment	Induced thyroid cytoarchitectural alterations and apoptosis, characterized by enlarged follicles, reduced follicle number, decreased follicular cell height, and increased BAX/Bcl2 ratio; these effects were attenuated by Aronia melanocarpa and, more markedly, by Hypericum perforatum	[285]
Lead	Wistar rats (6-week-old albino male)	Pb: 0.1, 0.5, 1, 3, 7, and 15 mg/kg/d	28 days	Increased serum T4, FT4, and TSH levels in a dose-dependent manner, with no significant changes in T3, FT3, anti-TPO, or anti-Tg levels, indicating hyperthyroidism-like thyroid disruption; toxicogenomic analysis implicated antioxidant defense- and Se-dependent pathways	[286]
	Male Wistar albino rats	Pb acetate: 30 mg/kg once every 2 days; Nano-Se: 0.5 mg/kg	14 weeks	Decreased serum fT3 and fT4 levels and increased TSH levels, accompanied by Pb accumulation in serum and thyroid tissue, oxidative-antioxidative imbalance, reduced ID1 expression, and upregulation of miR-224; these effects were attenuated by Se nanoparticles	[287]
	*Bufo gargarizans* embryos	Pb^2+^: 10, 50, 100, 1000, and 2000 μg/L	During embryogenesis	Disrupted growth and development in a concentration-dependent manner, induced morphological abnormalities, and altered thyroid hormone-related gene expression, with decreased Dio2, TRα, and TRβ and increased Dio3 at high concentrations, indicating thyroid-disrupting effects	[288]
	Wistar rat offspring	Lead acetate: 0.2% (1090 ppm) in dams’ drinking water; L-T4: 20, 50, or 100 μg/kg/d	Pb exposure via maternal milk from parturition to P21; L-T4 from P14 to P21	Induced hypothyroidism-like thyroid dysfunction, with decreased serum T3, T4, and TSH levels; these effects were attenuated by L-T4 treatment.	[289]
	Wistar rats	Lead acetate: 10 and 25 mg/kg	3 times/week for 14 days	Disrupted thyroid morphology and function, with reduced serum tT3, increased tT4, decreased thyroid NIS expression, reduced TSHr expression in females, morphometric alterations, vascular congestion, and increased collagen deposition	[290]
	Male Wistar rats	Lead acetate: 20 mg/kg; ascorbic acid: 0.2% in drinking water	15 days	Induced thyroid atrophy, reduced gland weight, decreased THs levels, and increased TSH levels; these alterations were attenuated by ascorbic acid	[291]
	*Bufo gargarizans* tadpoles	Pb^2+^: 50–1000 μg/L	From Gosner stage 26 to 42	Disrupted thyroid homeostasis, with thyroid follicular cell hyperplasia and colloid depletion, and dose-dependent dysregulation of Dio2, Dio3, TRα, and TRβ expression in the hind-limb, tail, and liver, leading to altered growth, metamorphosis, and skeletal ossifications	[292]

Abbreviations: Bax: Bcl-2-associated X protein; Bcl-2: B-cell lymphoma 2; CAT: Catalase; Cd: Cadmium; CdCl_2_: Cadmium chloride; CrVI: Hexavalent chromium; CXCL10: C-X-C motif chemokine ligand 10; Dio2: Deiodinase type 2; Dio3: Deiodinase type 3; E2: 17β-estradiol; ERα: Estrogen receptor alpha; ERK1/2: Extracellular signal-regulated kinase 1/2; FT3/fT3: Free triiodothyronine; FT4/fT4: Free thyroxine; GSH: Glutathione; ID1: Iodotyrosine deiodinase 1; MCP-1: Monocyte chemoattractant protein-1; MI: Myo-inositol; NaAsO2: Sodium arsenite; NF-κB: Nuclear factor kappa B; NIS: Sodium-iodide symporter; NLRP3: NOD-like receptor family pyrin domain-containing 3; Nrf2: Nuclear factor erythroid 2–related factor 2; Pb: Lead; PI3K: Phosphoinositide 3-kinase; PTEN: Phosphatase and tensin homolog; Se: Selenium; SOD: Superoxide dismutase; T3: Triiodothyronine; T4: Thyroxine; TAK-242: Toll-like receptor 4 inhibitor; Tg: Thyroglobulin; TgAb: Anti-thyroglobulin antibodies; THs: Thyroid hormones; TLR4: Toll-like receptor 4; TPO: Thyroperoxidase; TRα: Thyroid hormone receptor alpha; TRβ: Thyroid hormone receptor beta; TSH: Thyroid-stimulating hormone; TSHr: Thyroid-stimulating hormone receptor; TT3: Total triiodothyronine; TT4: Total thyroxine.

**Table 12:** In vivo evidence of endocrine-disrupting chemicals-induced alterations related to thyroid dysfunction and carcinogenesis.

EDCs	Animal Model	Dose	Exposure Time	Main Effects	Reference
BPA	Wistar rats (4–5-month-old female)	40 mg/kg	15 days	Increased thyroid H_2_O_2_ generation, reduced iodide uptake capacity, and decreased TPO activity, associated with oxidative stress	[252]
	BALB/c mice	20 mg/kg and 100 mg/kg	4 weeks	Thyroid follicular cell atypia, elevated FT3 levels, and increased growth of BCPAP xenograft, accompanied by upregulation of NOX4, p-AKT, and p-MEK expression	[253]
PCB118	Wistar rats (6–8 week-old male)	10, 100, and 1000 μg/kg/d	13 weeks	Reduced serum TH levels, induced ultrastructural and structural thyroid damage, accompanied by reduced Nis and Tg mRNA expression levels	[293]
PCB118	Wistar rats (6–8 week-old male)	10, 100, and 1000 μg/kg/d	13 weeks	Altered serum TH levels and induced structural thyroid damage, accompanied by reduced NIS levels, altered expression of genes involved in the mitochondrial respiratory chain, increased ROS generation, and activation of the JNK pathway	[262]
Perchlorate	Zebrafish	10 and 100 mg/L of Sodium Perchlorate	10, 30, 60, and 90 days	Induced thyroid histopathological alterations, including follicular cell hypertrophy, colloid depletion, hyperplasia, and angiogenesis	[280]
TBT	Pregnant Wistar rats and offspring	100 and 1000 ng/kg/d	From gestational day 7 to the end of lactation	Maternal TBT exposure induced dose-dependent thyroid morphological alterations in dams and offspring, with sex-specific endocrine effects, including increased T4 in female offspring and increased T3 in male offspring at birth	[294]
	Wistar rats (3-month-old female)	200 and 1000 ng/kg/d	40 days	Induced thyroid redox imbalance, with increased TPO activity and expression, reduced CAT and GPx activity, increased ERα mRNA expression, and increased interstitial collagen deposition, suggesting oxidative stress- and ERα-mediated thyroid alterations	[295]
	Wistar rats	100 ng/kg/d	15 days	Disrupted thyroid morphophysiology and HPT axis function in a sex-dependent manner, with stratified epithelium, cystic degeneration, and increased interstitial collagen deposition; T3 and T4 levels were reduced in males, whereas TSH levels, hepatic D1, and TRH mRNA expression were reduced in females, while hypothalamic D2 mRNA expression increased in males	[296]
	Zebrafish	10, 100, and 300 ng/L	6 weeks	Disordered the thyroid hormone system, induced immunotoxicity, and altered blood biochemical parameters, accompanied by dysregulation of genes related to thyroid hormone signaling and immune responses	[297]
	Male Wistar rats	100 ng/kg/d	15 days	Induced thyroid cytoarchitectural alterations and interstitial collagen deposition, accompanied by increased thyrocyte proliferation and higher SDHA expression, suggesting oxidative stress-related thyroid remodeling	[298]
	Female Wistar rats	200 and 1000 ng/kg/d	40 days	Disrupted the HPT axis, with increased TSH levels, decreased total T4 levels, altered TRH and Dio1 expression, and thyroid histomorphological alterations, suggesting interference with peripheral thyroid hormone metabolism	[299]

Abbreviations: AKT: Protein kinase B; BALB/c: Bagg albino mouse strain C; BPA: Bisphenol A; CAT: Catalase; D1/Dio1: Deiodinase type 1; D2: Deiodinase type 2; ERα: Estrogen receptor alpha; FT3: Free triiodothyronine; GPx: Glutathione peroxidase; H_2_O_2_: Hydrogen peroxide; HPT: Hypothalamic-pituitary-thyroid; JNK: c-Jun N-terminal kinase; MEK: Mitogen-activated protein kinase kinase; NIS/Nis: Sodium-iodide symporter; NOX4: NADPH oxidase 4; PCB: Polychlorinated biphenyl; ROS: Reactive oxygen species; SDHA: Succinate dehydrogenase complex flavoprotein subunit A; T3: Triiodothyronine; T4: Thyroxine; TBT: Tributyltin; Tg: Thyroglobulin; TH: Thyroid hormone; TPO: Thyroperoxidase; TRH: Thyrotropin-releasing hormone; TSH: Thyroid-stimulating hormone.

## Data Availability

No new data were created or analyzed in this study. Data sharing is not applicable to this article.
